# Trends in the prevalence and management of major metabolic risk factors for chronic disease over 20 years: findings from the 1998-2018 Korea National Health and Nutrition Examination Survey

**DOI:** 10.4178/epih.e2021028

**Published:** 2021-04-19

**Authors:** Yoonjung Kim, Sun Jin Nho, Gyeongji Woo, Hyejin Kim, Suyeon Park, Youngtaek Kim, Ok Park, Kyungwon Oh

**Affiliations:** 1 Division of Health and Nutrition Survey and Analysis, Bureau of Chronic Disease Prevention and Control, Korea Disease Control and Prevention Agency, Cheongju, Korea; 2 Public Health Medical Service Office, Chungnam National University Hospital, Daejeon, Korea

**Keywords:** Korea National Health and Nutrition Examination Survey, Obesity, Hypertension, Diabetes mellitus, Hypercholesterolemia

## Abstract

**OBJECTIVES:**

We aimed to explore trends in the prevalence and management of obesity, hypertension, diabetes, and hypercholesterolemia in Korean adults from 1998 to 2018 using data from the Korea National Health and Nutrition Examination Survey (KNHANES).

**METHODS:**

The study participants included 79,753 individuals aged ≥ 30 years who had participated in the health examination and health interview of the first (1998) to the seventh (2016-2018) KNHANES. The prevalence and management as well as annual percent change (APC) in chronic diseases were analyzed using SAS and the Joinpoint software program.

**RESULTS:**

The prevalence of obesity in men significantly increased from 26.8% in 1998 to 44.7% in 2018 (APC=1.9, p<0.001), whereas that in women decreased slightly from 30.5% in 1998 to 28.3% in 2018 (APC=-0.5, p<0.001). The prevalence of hypertension in men was 33.2% in 2018, with no significant change, whereas that in women slightly decreased to 23.1% in 2018 (APC=-0.9, p<0.001). The prevalence of diabetes in men increased slightly from 10.5% in 2005 to 12.9% in 2018 (APC=1.6, p<0.001), whereas that in women remained at approximately 8%, with no significant change. The prevalence of hypercholesterolemia in both men and women increased 3-fold in 2018 (20.9% in men [APC=8.2, p<0.001] and 21.4% in women [APC=7.1, p<0.001]) compared to that in 2005. The awareness rate, treatment rate, and control rate of hypertension and hypercholesterolemia increased 2-3 fold. Regarding diabetes, the treatment rate increased, but the control rate did not change.

**CONCLUSIONS:**

Over the past 20 years, the prevalence of obesity (in men), diabetes, and hypercholesterolemia has increased and management indicators, such as the awareness rate, treatment rate, and control rate of chronic diseases, have improved continuously.

## INTRODUCTION

Non-communicable diseases (NCDs), such as cardiocerebrovascular diseases, cancer, chronic respiratory diseases, and diabetes, are caused by environmental risk factors and poor lifestyles [1]. In 2016, 41 million people, accounting for 70% of the world’s deaths, died due to NCDs. Among the NCDs, cardiocerebrovascular diseases (17.9 million), cancer (9 million), chronic respiratory diseases (3.8 million), and diabetes (1.6 million) were the most prevalent causes of death [2]. The World Health Organization (WHO) established the ‘WHO Global NCD Action Plan 2013-2020’ to achieve the global target of a 25% relative reduction in the risk of premature mortality from NCDs and urged countries to establish public health policies and monitor the process indicators toward these national targets accordingly [3]. In Korea, the top 10 causes of death, accounting for 69.1% of total deaths, are malignant neoplasms, heart disease pneumonia, cerebrovascular diseases, intentional self-harm, diabetes, liver diseases, chronic lower respiratory diseases, Alzheimer’s disease, and hypertensive diseases, with chronic diseases being strong contributors [4]. Therefore, for the intensive management of the four chronic diseases (cardiocerebrovascular diseases, diabetes, chronic respiratory diseases, and cancer), the Health Plan 2020 (HP2020) established targets to reduce the prevalence of hypertension, diabetes, hypercholesterolemia, and obesity and improve the management of these diseases. The Korea National Health and Nutrition Examination Survey (KNHANES) monitored the progress toward attainment of the HP2020 goals [5].

This study analyzed trends in the prevalence and management of obesity, hypertension, diabetes, and hypercholesterolemia in Korean adults using data from the KNHANES from 1998 to 2018.

## MATERIALS AND METHODS

### Subjects

The KNHANES was conducted to assess the health and nutritional status in the Korean population [6]. This survey uses a two-stage stratified cluster sampling method to select approximately 200 primary sampling units (PSUs) consisting of 20-23 households per PSU. All members aged ≥ 1 year within the sampled households are eligible for the survey.

Individuals aged ≥ 30 years who participated in the health examination and health interview of the KNHANES were included in the analysis. Participants with a fasting time of < 8 hours (n=4,343) and pregnant women (n=303) were excluded in the hypercholesterolemia and obesity statistics analysis, respectively.

### Health examination

The health examinations in the first (1998), second (2001), third (2005), and fourth (2007) KNHANES were conducted at a center located close to the survey district. From 2008, health examinations were conducted in the mobile examination center (MEC) by trained medical personnel from the Korea Disease Control and Prevention Agency (KDCA, formerly Korea Centers for Disease Control and Prevention). The height to the nearest 0.1 cm was measured using wall-mounted stadiometers (SECA 225; Seca GmbH, Hamburg, Germany), and weight to the nearest 0.1 kg was measured using a portable electronic scale. Blood pressure was measured three times on the right arm of the subject in a sitting position after 5 minutes of rest using a mercury sphygmomanometer (Baumanometer; Baum, Copiague, NY, USA) by a trained nurse (measured twice and averaged in 1998 and 2001, measured thrice and averaged the second and third measurements from 2005). Blood pressure and body measurements were performed for quality assurance and control in academic societies [7,8].

To calculate the prevalence of diabetes and hypercholesterolemia, the blood was collected from each participant in the morning after fasting for at least 8 hours. After blood collection, processing, storing in MEC, all specimens were shipped to the clinical laboratory at 2-8°C on the survey day for analysis.

The equipment and reagents for analyzing blood glucose and cholesterol were changed due to a change of laboratory in 2005 and 2008, and the blood collection tubes for blood glucose measurement were switched from sodium fluoride tube to serum separating tube in 2014 [9]. From 2008, total cholesterol and fasting blood glucose were analyzed by the enzymatic method (Hitachi Automatic Analyzer 7600; Hitachi, Tokyo, Japan) and hemoglobin Alc were analyzed using high-performance liquid chromatography (Tosoh G8; Tosoh, Tokyo, Japan). From 2005, the quality control of laboratory testing was conducted in the Korean Society for Laboratory Medicine [10]. The history of diagnosis and medication were investigated through interviews.

### Definition of chronic disease prevalence and management indicators

Body mass index (BMI) was calculated using height and weight, and BMI ≥ 25 kg/m^2^ was classified as obesity according to the Korean Society for the Study of Obesity [11]. According to the Korean Society of Hypertension, hypertension was defined as systolic blood pressure ≥ 140 mmHg, diastolic blood pressure ≥ 90 mmHg, or taking antihypertensive medication [12]. Based on the Korean Diabetes Association, diabetes was defined as fasting blood glucose ≥ 126 mg/dL or diagnosis of diabetes by a physician or taking hypoglycemic drugs or usage of insulin [13]. Based on the standards of the Korean Society of Lipid and Atherosclerosis, hypercholesterolemia was defined as total cholesterol of ≥ 240 mg/dL or taking cholesterol-lowering drugs [14].

Regarding the prevalence and management indicators for diabetes and hypercholesterolemia, in the 1998 and 2001, it was difficult to reconfirm the fasting time before blood collection and hemoglobin Alc, and there were no questions asked on the taking hypercholesterolemia medications; therefore, these data were included from 2005.

The awareness rate of hypertension, diabetes, and hypercholesterolemia was defined as having had a lifetime diagnosis by a physician, and the treatment rate was defined as currently taking medication among participants who were defined as having hypertension, diabetes, or hypercholesterolemia. The control rate was defined as reaching of the goals among those receiving treatment (hypertension: systolic blood pressure < 140 mmHg, diastolic blood pressure < 90 mmHg, diabetes: hemoglobin Alc < 6.5%, hypercholesterolemia: blood cholesterol < 200 mg/dL).

### Statistical analysis

All analyses were performed using SAS version 9.4 (SAS Institute Inc., Cary, NC, USA) and Joinpoint Regression Program version 4.1.1.1 (US National Cancer Institute, Bethesda, MD, USA). To represent the Korean population, sampling weights assigned to participants were applied to all analyses. Sampling weights were generated by considering the complex sample design, the non-response rate of the target population, and post-stratification. To adjust for differences in the results of changes in the age structure of each year, age-standardized prevalence was calculated using age-specific and gender-specific structures of the estimated population based on the 2005 Population Projections for Korea.

Trends in the prevalence of obesity, hypertension, diabetes, and hypercholesterolemia according to gender and household income level were age standardized using SAS (PROC SURVEYREG). The prevalence according to age and management indicators were crude rates and were calculated using SAS (PROC SURVEYMEANS). Data on management indicators were combined as 3-year data to obtain an acceptable level of reliability. The estimates and their standard errors obtained from SAS were input into Joinpoint program, which was estimated by setting the Joinpoint as 0 or 1, and the APC was calculated accordingly. The APC verified that the annual rate of change was “0” under a significance level of 0.05, and the Monte Carlo method in Joinpoint Regression Program was used to test the statistical significance of the optimal model.

### Ethics statement

This study was approved by the Institutional Review Board of the KDCA (2007-2014, 2018). For certain year (2015-2017), ethical approval was waived by the Act (Article 2, Paragraph 1) and Enforcement Regulation (Article 2, Paragraph 2, item 1) of Bioethics and Safety Act.

## RESULTS

### Subjects’ characteristics

Table 1 shows the general characteristics of the participants of the first (1998) to the seventh (2016-2018) KNHANES. The number of participants aged ≥ 30 years was 79,753. The percentage of participants in their 20s-40s decreased over the 20 years, whereas the percentage of those in their ≥ 50s increased between 1998 and 2018.

### Obesity

The prevalence of obesity (age-standardized, aged ≥ 30 years) significantly increased from 26.8% in 1998 to 44.7% in 2018 in men (APC =1.9, p<0.001), whereas it slightly decreased from 30.5% in 1998 to 28.3% in 2018 in women (APC=-0.5, p<0.001). During the 20 years, especially after 2005, the prevalence of obesity significantly increased in men in their 30s and ≥ 60s, whereas it significantly decreased in women in their 50s and 60s (Table 2). The prevalence of obesity according to the household income level in men increased in all the groups, whereas that in women decreased in the upper middle household income level groups; it significantly decreased after 2007 (APC=-2.3, p<0.001), especially in the high household income group. In women, the difference in the prevalence of obesity between the high-household and low-household income groups increased by eight times from -2.1%p (28.0 and 30.1%) in 1998 to -17.5%p (18.0 and 35.5%) in 2018 (Table 2).

### Hypertension

The prevalence of hypertension (age-standardized, aged ≥ 30 years) in men did not significantly change over the 20 years, with rates of 32.4% in 1998 to 33.2% in 2018, but that in women decreased slightly from 26.8% in 1998 to 23.1% in 2018 (APC=-0.9, p<0.001). The prevalence of hypertension according to age in men in their 30s and 40s significantly increased after 2009 and 2007, respectively (APC =3.5, 1.8, p<0.001), whereas that in women in their 50s and 60s significantly decreased after 2005 and 2011, respectively (APC=-1.8, -2.5, p<0.001), and men and women in their 70s significantly increased after 2005 (APC=2.1, 1.1, p<0.001) (Table 3). The prevalence of hypertension according to household income level in men was not significantly different in all the groups during the 20 years, whereas that in women significantly decreased in the upper middle household income groups until 2016. The difference in the prevalence of hypertension between the high-household and low-household income groups increased over 20 years in both men and women; the difference in prevalence increased by three times from -2.7%p (25.1 and 27.8%) in 1998 to -8.5%p (17.4 and 25.9%) in 2018, especially in women.

The awareness rate of hypertension significantly increased from 23.5% in 1998 to 69.1% in 2016-2018; the treatment rate of hypertension increased from 20.4% in 1998 to 65.3% in 2016-2018, and the control rate of hypertension among participants with hypertension who received treatment significantly increased from 23.8% in 1998 to 73.1% in 2017-2018 (APC=9.2, 11.1, 6.2, p<0.001) (Table 4), indicating that when hypertension was diagnosed, most patients received treatment, and their blood pressure was controlled by the treatment. The awareness rate (19.8 and 44.8% in 2016-2018) and the treatment rate (16.9 and 38.2% in 2016-2018) in the 30s and 40s age groups were lower than those in the other age groups; in particular, the rates in the 30s age group were < 20% [15].

### Diabetes

The prevalence of diabetes (age-standardized, aged ≥ 30 years) in men significantly increased from 10.5% in 2005 to 12.9% in 2018 (APC=1.6, p<0.001), and this trend was more prominent since 2009 (APC=1.9, p<0.001), whereas that in women remained at around 8%, with no significant changes (APC=0.5). The prevalence of diabetes according to age in men aged ≥ 70 years significantly increased before 2010 (APC =8.1, p<0.001) and that in women clearly increased before 2016 (APC=3.8, p<0.001). The prevalence of diabetes according to household income level increased in both men and women low-household income groups (APC=3.2, 2.8, p<0.001). The difference in prevalence between the high-household and low-household income groups was 2.4%p (12.2 and 9.8%) in 2005 to -4.4%p (10.9 and 15.3%) in 2018 in men and 1.3%p (7.4 and 6.1%) in 2005 to -5.4%p (5.2 and 10.6%) in women, with an increasing gap observed between the high household and low household income levels (Table 5).

The awareness rate of diabetes significantly increased only in women (APC=1.6, p<0.001), although the treatment rate of diabetes increased in both men and women (APC=4.7, 5.5, p<0.001). The control rate of diabetes among participants who received treatment showed no significant changes in either gender (Table 4). Among participants in their 30s and 40s, the awareness rate (33.6 and 47.9% in 2016-2018) and treatment rate (28.5 and 42.4% in 2016-2018) of diabetes were higher than those of hypertension or hypercholesterolemia, but they were < 50% [15].

### Hypercholesterolemia

The prevalence of hypercholesterolemia (age-standardized, aged ≥ 30 years) in men significantly increased from 7.3% in 2005 to 20.9% in 2018, showing a 13.6%p increase (APC=8.2, p<0.001), with a significant increase after 2014 (APC=9.9, p<0.001). The prevalence of hypercholesterolemia in women increased from 8.4% in 2005 to 21.4% in 2018, showing a 13.0%p increase (APC=7.1, p<0.001), and it specifically increased after 2008 (APC=6.8, p<0.001). The prevalence of hypercholesterolemia in men and women increased in all age groups (p<0.001), with a particularly clear trend of increase before 2016 in women aged ≥ 40 years. Among participants aged < 50 years, the prevalence of hypercholesterolemia in men was 1.5 times higher than that in women; however, among those aged ≥ 50 years, the prevalence in women was 1.5 times higher than that in men. In addition, the prevalence of hypercholesterolemia in all groups according to the household income level significantly increased during the 20-year period (Table 6).

The awareness rate of hypercholesterolemia increased from 24.0% in 2005 to 60.1% in 2016-2018 (APC=16.1, p<0.001), and the treatment rate of hypercholesterolemia increased from 17.3% in 2005 to 50.3% in 2016-2018 (APC=22.0, p<0.001) (Table 4). The control rate among participants who received treatment increased from 61.8% in 2005 to 82.7% in 2016-2018 in women only (APC=4.5, p<0.001). The rate of awareness (18.0 and 40.3% in 2016-2018,) and the treatment (10.6 and 27.9% in 2016-2018,) in hypercholesterolemia among the 30s and 40s age groups was < 50%, and in particular, the treatment rate among those in their 30s was the lowest among that of other chronic diseases [15].

## DISCUSSION

Analyses of the 20-year trend in the prevalence and management of metabolic risk factors for cardiocerebrovascular disease using the KNHANES data revealed an increase in the prevalence of obesity, diabetes, and hypercholesterolemia. Further, the awareness, treatment, and control rates of hypertension and hypercholesterolemia had all improved, only the treatment rate of diabetes had improved (Table 4).

The prevalence of obesity (age-standardized, aged ≥ 30 years) in men markedly increased in all age groups over 20 years (26.8% in 1998, 44.7% in 2018, APC=1.9) and that in women slightly decreased (30.5% in 1998, 28.3% in 2018, APC=-0.5). Hence, the HP2020 goals of 37.0% in men and 27.0% in women were not achieved. Because the obesity criteria are different for each country, BMI ≥ 25 kg/m^2^ and aged ≥ 20 years were used for comparison between the countries in this study. The prevalence of obesity in Korea (age-standardized, aged ≥ 19 years in 2018) was 42.8% in men and 25.5% in women, which was half of that in the United States (age-standardized, aged ≥ 20 years in 2013-2016; 74.6% in men and 67.4% in women) [16]. However, the prevalence of obesity in Korea was higher than that in Japan (age-standardized, aged ≥ 20 years in 2018; 31.6% in men and 19.6% in women) [17]. It is concerning that the prevalence of obesity in men continues to increase, with a prevalence of 50% in the 30s and 40s age groups and that the prevalence of obesity in women in their 20s and 30s is increasing. Considering that obesity is a strong risk factor for chronic diseases, it is necessary to actively promote the guidelines for the management of obesity established in 2018 in Korea through network of collaborating in various fields, such as nutrition, physical activity, and healthcare.

The prevalence of hypertension (age-standardized, aged ≥ 30 years) in men did not change significantly over 20 years, with a prevalence of 33.2% in 2018, whereas that in women slightly decreased to 23.1% in 2018, which is above the HP2020 goal of 23.0%. The prevalence of hypertension in Korea (age-standardized, aged ≥ 19 years in 2018) was 27.6% in men and 18.3% in women, which was lower than that in the United States (age standardized, aged ≥ 20 years in 2015-2016; 31.3% in men and 28.7% in women) and similar to that in Japan (age-standardized, aged ≥ 20 years in 2018; 28.0% in men and 19.0% in women) [16,17]. The prevalence of hypertension did not significantly increase compared to that of other chronic diseases. Despite worsening of obesity and physical activity being major risk factors [18], policies promoted after 1999 (Healthy Lifestyle Practice [1999], National Chronic Diseases Management [2000], and Regional Cardiocerebrovascular Disease Center [2005]) resulted in increased awareness regarding the importance of blood pressure management and lifestyle improvement, leading to early detection of hypertension and increased treatment [19-21]. However, despite follow-up interventions, such as customized home visiting health care service (2007), registration and management of patients with hypertension or diabetes (2007), national health check-up by life cycle (2007), follow-up management on health check-up (2008), chronic disease management in the clinic (2012), and sodium reduction (2012), management indicators have not changed since 2007. This may be related to individuals in their 30s and 40s who are maintaining a low rate of awareness and treatment rates compared to other age groups, and it has been suggested that an interest in hypertension and encouraging periodic health check-ups are required to improve management indicators in these age groups [22].

The prevalence of diabetes (age-standardized, aged ≥ 30 years) in men slightly increased from 10.5% in 2005 to 12.9% in 2018, whereas that in women remained at 8%, with no significant changes. Hence, the HP2020 goal of 9.7% was not achieved. The prevalence of impaired fasting glucose, the intermediate stage for cardiocerebrovascular diseases, was 33.2% in men and 23.4% in women in 2018, which was 2-3 times higher than that of diabetes. In the United States and Finland, diabetes prevention programs are provided as lifestyle modification programs to prevent pre-diabetes, such as impaired fasting glucose or impaired glucose tolerance [23,24]. In Korea, considering the status and trend of pre-diabetes, it is necessary to not only manage diabetes but also actively promote preventive programs to avoid the transition from pre-diabetes to diabetes.

The prevalence of hypercholesterolemia (age-standardized, aged ≥ 30 years) in 2018 was 20.9% in men and 21.4% in women, and it did not meet the HP2020 goal of 13.5%. The reason why the prevalence of hypercholesterolemia among 30s and 40s age groups is approximately twice as high in men (15.1 and 22.9%, respectively, in 2018) as that in women (9.2 and 10.5%, respectively, in 2018) may be related to the high smoking rate in men in their 30s and 40s (39.9 and 44.1%), which is a major risk factor for hypercholesterolemia [15,25]. Furthermore, men in these age groups had a higher proportion of excessive energy and fat intake than those in other groups (10.6 and 9.6% in men; 5.8 and 4.7% in women) and they had a lower proportion of healthy eating practices (36.3 and 37.4% in men; 52.1 and 55.6% in women) than those in other groups [15]. Among participants in the ≥ 50s age groups, the prevalence of hypercholesterolemia was higher in women than in men, possibly because of the effect of hormonal changes due to menopausal status on blood cholesterol metabolism in the body [26].

When comparing the prevalence of chronic diseases according to gender and age, the prevalence of obesity in men was the highest in those in their 30s, and the prevalence of diabetes and hypercholesterolemia rapidly increased from 40 years of age. Men in their 30s and 40s have a high proportion of excessive energy and fat intake (10.6 and 9.6%) and binge drinking (55.9 and 59.3%), and have low rate of aerobic physical activity (55.3 and 48.8%) [15], which may have affected the increase in the prevalence of diabetes and hypercholesterolemia in men aged ≥ 40 years. In a study analyzing the effects of age, period, and cohort based on the KNHANES data [27], the prevalence of obesity and hypercholesterolemia tended to increase in birth cohorts after the 1970s. The cohort born in the 1970s was in their 40s in 2018. Hence, with increasing age, the prevalence of chronic diseases, such as obesity and hypercholesterolemia, is expected to increase in this group.

The management of chronic diseases improved overall, but the control rate of diabetes remained at 25%, with no significant change, and was lower than that of other chronic diseases. In this regard, an increase in the elderly population, an increase in the duration of the disease, low adherence to a healthy lifestyle, and low compliance to diabetes medication could be related factors for this occurrence [28-30]. The awareness rate of all chronic diseases was low in the 30s and 40s age groups, suggesting that a program to raise the awareness rate in the young age groups and create a healthy lifestyle ahead of onset of chronic disease. In addition, the difference in the prevalence of chronic diseases between the high-income and low-income groups in women increased during the 20-year period (15.4%p for obesity [-2.1%p in 1998 and -17.5%p in 2018], 5.8%p for hypertension [-2.7%p in 1998 and -8.5%p in 2018], and 6.7%p for diabetes [1.3%p in 2005 and -5.4%p in 2018]), thus supporting the need for programs to reduce the health gap.

According to the KNHANES data, the prevalence of obesity and hypercholesterolemia has increased over the past 20 years, and trends in the management of hypertension, hypercholesterolemia, and diabetes flattened in recent years. In the future, policies should be promoted to decrease the prevalence of chronic diseases, reduce health disparities, and improve chronic disease management indicators.

## Figures and Tables

**Table 1.** Characteristics of health examination participants of Korea National Health and Nutrition Exami nation Survey

Characteristics		1998	2001	2005	2007-2009	2010-2012	2013-2015	2016-2018
Total (≥30 yr)		6,469	5,500	4,818	15,126	16,431	15,042	16,367
Men		2,938 (45.4)	2,410 (43.8)	2,065 (42.9)	6,389 (42.2)	7,033 (42.8)	6,410 (42.6)	7,121 (43.5)
Age (yr)								
	30-39	1,968 (30.4)	1,667 (30.3)	1,226 (25.4)	3,498 (23.1)	3,403 (20.7)	2,758 (18.3)	2,920 (17.8)
	40-49	1,630 (25.2)	1,525 (27.7)	1,346 (27.9)	3,425 (22.6)	3,294 (20.0)	3,105 (20.6)	3,415 (20.9)
	50-59	1,260 (19.5)	949 (17.3)	918 (19.1)	2,907 (19.2)	3,534 (21.5)	3,342 (22.2)	3,523 (21.5)
	60-69	1,007 (15.6)	804 (14.6)	811 (16.8)	2,790 (18.4)	3,146 (19.1)	2,907 (19.3)	3,229 (19.7)
	≥70	604 (9.3)	555 (10.1)	517 (10.7)	2,506 (16.6)	3,054 (18.6)	2,930 (19.5)	3,280 (20.0)
Household income^1^								
	Low	1,246 (19.3)	956 (18.5)	1,003 (21.0)	2,942 (20.0)	3,233 (20.0)	2,994 (20.0)	3,240 (19.9)
	low-middle	1,220 (18.9)	1,091 (21.1)	934 (19.6)	2,972 (20.2)	3,263 (20.1)	2,965 (19.9)	3,285 (20.1)
	Middle	1,358 (21.0)	1,001 (19.4)	929 (19.4)	2,904 (19.8)	3,239 (20.0)	2,996 (20.1)	3,262 (20.0)
	Middle-high	1,355 (20.9)	995 (19.3)	969 (20.3)	2,960 (20.1)	3,205 (19.8)	3,023 (20.2)	3,263 (20.0)
	High	1,290 (19.9)	1,121 (21.7)	942 (19.7)	2,918 (19.9)	3,254 (20.1)	2,957 (19.8)	3,255 (20.0)
Area of residence								
	Urban area (dong)	3,970 (61.4)	4,151 (75.5)	3,629 (75.3)	10,806 (71.4)	12,773 (77.7)	11,883 (79.0)	13,132 (80.2)
	Rural area	2,499 (38.6)	1,349 (24.5)	1,189 (24.7)	4,320 (28.6)	3,658 (22.3)	3,159 (21.0)	3,235 (19.8)

Values are presented as number (weighted %).

1 Calculated as monthly household income divided by square root of the number of persons in the household, categorized into quantiles according to age and gender.

**Table 2.** Trends in the prevalence of obesity among Koreans aged ≥30 years^1^

Variables			1998	2001	2005	2007	2008	2009	2010	2011	2012	2013	2014	2015	2016	2017	2018	Difference (1998-2018)	APC^2^ 1998-2018	APC and year of any significant change in trend slope		
																				Before	Change year	After
Total (≥30 yr)			29.1	32.7	34.8	34.6	32.9	34.0	33.9	34.2	35.4	34.6	32.9	36.0	37.0	35.5	36.9	7.8	0.9	1.7	2005	0.5^***^
Men																						
	Age (yr)																					
		Total	26.8	33.6	37.6	37.8	36.6	37.8	38.7	37.7	38.0	40.1	39.5	41.8	43.3	42.4	44.7	17.9	1.9^***^	3.6^***^	2005	1.4^***^
		30-39	28.4	35.0	38.1	41.7	38.2	38.5	42.3	40.7	40.6	47.1	43.9	43.6	45.4	46.7	51.3	22.9	2.4^***^	3.4	2005	2.0^***^
		40-49	33.3	39.0	41.1	37.9	41.1	41.9	41.3	42.6	45.0	41.5	39.6	45.6	49.0	44.7	47.5	14.2	1.4^***^	1.2^***^	2014	2.4
		50-59	28.3	32.4	41.0	41.7	39.6	43.4	36.8	34.7	33.2	40.8	41.5	40.3	39.7	44.3	40.9	12.6	1.4^***^	4.9	2005	0.3
		60-69	20.0	28.0	31.0	34.6	29.8	32.2	37.8	34.1	33.5	29.3	36.9	38.3	39.7	36.7	38.1	18.1	2.4^***^	5.5	2005	1.6^***^
		≥70	8.0	23.0	27.4	21.4	21.0	19.9	24.5	23.7	23.0	26.2	24.0	32.1	30.3	25.3	30.6	22.6	2.9^***^	6.2	2005	2.4^***^
	Household income^3^																					
		Low	24.1	32.7	33.9	34.6	34.4	34.7	32.0	28.9	32.1	35.3	39.8	44.0	44.8	45.4	39.0	14.9	2.5^***^	1.5	2011	4.4^***^
		Low-middle	25.7	31.1	34.3	34.4	36.4	37.7	43.0	38.1	44.8	42.9	41.5	47.2	39.7	43.2	49.7	24	2.7^***^	3.6^***^	2010	1.8
		Middle	25.9	34.9	35.9	39.0	40.6	37.0	35.0	40.9	36.7	40.3	39.6	40.4	44.8	38.1	43.5	17.6	1.8^***^	4.2	2005	0.9
		Middle-high	27.3	34.1	40.5	45.0	35.0	38.3	39.2	44.7	37.7	37.2	39.3	39.2	40.9	46.1	49.5	22.2	1.9^***^	1.5^***^	2016	8.7
		High	30.5	35.0	42.1	38.7	39.0	41.2	44.1	36.9	40.1	45.4	37.5	40.4	45.8	39.8	41.3	10.8	1.2^***^	4.0	2005	0.2
Women																						
	Age (yr)																					
		Total	30.5	32.1	31.3	30.3	28.4	29.5	28.5	30.1	32.2	28.2	25.7	29.6	30.0	27.7	28.3	-2.2	-0.5^***^	-0.3	2005	-0.6
		30-39	20.9	19.1	19.0	12.8	17.0	19.6	19.0	21.7	23.7	17.9	18.6	21.1	21.7	18.3	22.6	1.7	0.2	-1.8	2007	2.0^***^
		40-49	29.8	33.6	29.0	26.6	27.5	27.2	26.7	27.9	33.2	25.7	22.3	25.4	28.7	25.6	25.7	-4.1	-1.0^***^	-0.8	2012	-1.3
		50-59	42.7	40.8	43.1	43.1	35.3	36.7	33.8	36.7	34.9	33.7	29.3	36.2	32.5	31.7	29.3	-13.4	-1.8^***^	-0.4	2005	-2.3^***^
		60-69	38.6	46.6	47.1	56.4	43.8	41.4	43.3	43.0	43.1	42.7	36.6	41.7	40.7	39.3	35.5	-3.1	-0.8	2.7	2005	-2.0^***^
		≥70	29.4	33.4	34.0	37.9	34.3	38.1	34.4	33.5	36.1	38.6	37.3	40.8	42.2	41.0	43.0	13.6	1.7^***^	1.0^***^	2011	2.7^***^
	Household income^3^																					
		Low	30.1	36.0	34.3	29.5	35.9	35.0	32.3	37.1	38.4	33.2	29.9	41.6	37.8	36.1	35.5	5.4	0.7	0.9	2016	-2.4
		Low-middle	31.1	35.7	34.4	32.4	31.9	30.8	28.1	29.2	36.8	32.5	27.1	29.1	31.8	30.6	32.3	1.2	-0.4	-0.6	2015	1.7
		Middle	33.0	32.5	31.8	32.0	28.4	31.5	30.0	33.8	27.9	28.6	29.6	28.0	29.3	26.7	30.9	-2.1	-0.7^***^	-0.8^***^	2016	2.0
		Middle-high	30.0	30.1	31.4	26.4	25.8	25.7	28.7	22.6	29.7	26.0	25.6	29.6	27.6	24.2	22.3	-7.7	-1.0^***^	-0.7	2016	-8.0
		High	28.0	27.3	24.9	30.1	20.9	24.9	23.6	26.0	26.0	19.7	17.1	20.5	23.8	21.1	18.0	-10	-1.8^***^	-1.0	2007	-2.3^***^

Values are presented as weighted %.

1 Age-standardized prevalence was calculated using the 2005 Population Projections for Korea.

2 The annual percent change (APC) is significantly different from 0.

3 Calculated as monthly household income divided by square root of the number of persons in the household, categorized into quantiles according to age and gender.

*** p<0.001.

**Table 3.** Trends in the prevalence of hypertension among Koreans aged ≥30 years^1^

Variables			1998	2001	2005	2007	2008	2009	2010	2011	2012	2013	2014	2015	2016	2017	2018	Difference (1998-2018)	APC^2^ 1998-2018	APC and year of any significant change in trend slope		
																				Before	Change year	After
Total (≥30 yr)			29.8	28.5	28.0	24.5	26.2	26.3	26.8	28.4	28.9	27.2	25.4	27.8	29.1	26.9	28.3	-1.5	-0.2	-1.3	2007	0.6
Men																						
	Age (yr)																					
		Total	32.4	33.2	31.5	26.8	28.1	30.3	29.3	32.8	32.1	32.4	29.7	32.6	35.0	32.3	33.2	0.8	0.3	-1.1	2008	1.4^***^
		30-39	18.6	17.6	14.1	13.3	15.2	11.5	12.6	14.6	15.5	15.8	13.6	15.9	16.9	17.9	17.1	-1.5	-0.6	-3.3^***^	2009	3.5^***^
		40-49	30.5	28.6	27.8	19.8	23.5	25.2	22.4	31.2	26.9	28.5	26.9	28.4	30.8	26.9	29.1	-1.4	0.0	-2.6	2007	1.8^***^
		50-59	42.0	40.0	44.1	36.0	35.9	40.2	41.0	38.0	38.7	41.3	36.8	38.4	42.3	39.4	40.2	-1.8	-0.2	-0.9	2008	0.3
		60-69	43.8	56.8	53.9	43.4	43.0	52.1	52.5	53.5	55.3	48.5	45.5	52.1	55.9	47.6	47.5	3.7	0.1	0.5	2016	-5.8
		≥70	48.8	52.5	43.9	51.9	48.4	58.5	50.1	58.9	58.1	59.0	55.8	61.7	64.2	61.6	65.1	16.3	1.7^***^	-0.2	2005	2.1^***^
	Household income^3^																					
		Low	34.8	37.2	35.8	32.8	29.9	29.0	28.7	31.2	30.5	33.3	28.1	39.2	38.3	35.0	35.4	0.6	0.1	-1.8^***^	2010	3.0^***^
		Low-middle	33.2	31.1	31.2	28.7	28.7	33.0	29.3	31.7	33.5	31.1	31.6	32.3	31.1	32.4	37.6	4.4	0.4	-0.1	2016	8.9
		Middle	31.1	33.8	26.8	26.6	28.1	27.3	31.2	39.5	33.2	33.5	28.7	29.2	32.6	27.4	30.4	-0.7	0.0	0.6	2012	-1.7
		Middle-high	30.3	33.0	30.8	25.5	27.1	30.7	29.1	29.3	34.5	31.7	32.7	31.9	38.1	32.7	32.1	1.8	0.7	-1.1	2007	1.9^***^
		High	33.3	30.4	32.5	23.9	26.5	31.0	27.9	31.2	29.5	31.7	27.5	30.8	35.2	33.9	30.0	-3.3	0.1	-1.8	2007	1.4^***^
Women																						
	Age (yr)																					
		Total	26.8	25.3	23.8	21.7	23.8	22.1	23.8	23.6	25.2	22.1	20.9	22.9	22.9	21.3	23.1	-3.7	-0.9^***^	-1.7	2005	-0.5
		30-39	6.2	5.4	3.1	1.5	4.3	2.9	1.6	3.4	3.2	3.7	1.1	1.6	3.3	4.2	5.8	-0.4	-2.7	-6.0^***^	2015	40.4
		40-49	19.6	15.2	11.2	11.4	15.0	11.0	11.6	10.8	18.1	10.7	8.8	13.0	12.4	11.3	11.9	-7.7	-2.4^***^	-5.8	2005	-0.5
		50-59	37.2	33.2	38.4	31.1	32.6	30.5	33.7	29.7	30.4	30.6	27.4	29.5	30.8	24.9	29.1	-8.1	-1.4^***^	-0.7	2005	-1.8^***^
		60-69	50.5	57.5	53.6	48.2	50.2	49.1	58.3	57.1	52.8	48.8	51.1	51.5	46.2	46.3	44.6	-5.9	-0.6	0.3	2011	-2.5^***^
		≥70	63.4	61.5	61.3	65.4	61.5	65.9	68.2	71.5	71.6	64.3	68.7	71.3	72.5	66.8	73.7	10.3	0.9^***^	0.1	2005	1.1^***^
	Household income^3^																					
		Low	27.8	25.4	25.8	24.9	26.0	23.8	26.6	24.0	26.2	21.8	24.6	24.9	27.5	24.8	25.9	-1.9	-0.3	-0.8^***^	2013	1.6
		Low-middle	25.7	25.1	24.4	20.7	24.3	21.6	22.7	26.2	27.6	23.9	24.8	28.8	21.5	22.6	26.5	0.8	-0.1	-1.1	2007	0.7
		Middle	29.7	26.2	23.8	22.1	25.0	22.9	23.0	23.9	26.0	26.7	19.2	20.0	21.5	23.4	23.5	-6.2	-1.2^***^	-1.5^***^	2016	5.0
		Middle-high	25.9	26.5	23.4	19.2	21.5	19.7	23.0	22.2	22.8	18.5	18.1	20.6	22.7	17.5	22.3	-3.6	-1.4^***^	-1.8^***^	2014	1.8
		High	25.1	23.3	22.1	22.3	22.3	21.8	24.5	21.1	22.4	20.0	18.4	20.4	21.0	18.8	17.4	-7.7	-1.4^***^	-1.1^***^	2016	-7.1

Values are presented as weighted %.

1 Age-standardized prevalence was calculated using the 2005 Population Projections for Korea.

2 The annual percent change (APC) is significantly different from 0.

3 Calculated as monthly household income divided by square root of the number of persons in the household, categorized into quantiles according to age and gender.

*** p<0.001.

**Table 4.** Trends in the awareness, treatment, and control rates of hypertension, diabetes, and hypercholesterolemia among Koreans aged ≥30 years

Variables			1998	2001	2005	2007-2009	2010-2012	2013-2015	2016-2018	Difference (1998 to 2018)	APC^1^ 1998-2018
Hypertension											
	Awareness										
		Total	23.5	34.1	57.1	66.3	65.9	67.3	69.1	45.6	9.2^***^
		Men	17.3	26.8	48.4	58.4	56.9	60.1	64.0	46.7	12.1^***^
		Women	30.0	40.8	67.2	75.0	75.7	75.4	75.2	45.2	6.1
	Treatment										
		Total	20.4	32.7	49.6	60.3	60.7	63.6	65.3	44.9	11.1^***^
		Men	13.8	25.2	39.4	51.1	51.4	55.4	59.7	45.9	14.3^***^
		Women	27.5	39.5	61.4	70.3	70.7	72.7	72.0	44.5	7.5^***^
	Control										
		Total	23.8	37.6	54.9	69.3	69.1	72.0	73.1	49.3	6.2^***^
		Men	23.5	30.2	51.1	68.7	71.8	72.4	75.8	52.3	7.0^***^
		Women	24.0	42.0	57.8	69.7	67.0	71.7	70.5	46.5	4.6
Diabetes											
	Awareness										
		Total	-	-	68.3	72.6	72.7	70.5	71.5	3.2	0.0
		Men	-	-	67.0	70.2	69.5	66.6	66.7	-0.3	-1.5
		Women	-	-	69.9	75.5	76.7	75.5	77.7	7.8	1.6^***^
	Treatment										
		Total	-	-	49.0	57.5	63.9	63.3	66.2	17.2	4.9^***^
		Men	-	-	44.8	53.6	60.8	59.1	61.7	16.9	4.7^***^
		Women	-	-	54.2	62.0	67.7	68.9	71.9	17.7	5.5^***^
	Control										
		Total	-	-	22.0	24.6	25.0	22.3	25.8	3.8	0.7
		Men	-	-	25.1	27.1	23.7	23.2	25.1	0.0	-2.0
		Women	-	-	19.0	22.0	26.5	21.3	26.5	7.5	3.5
Hypercholesterolemia											
	Awareness										
		Total	-	-	24.0	38.8	47.4	57.7	60.1	36.1	16.1^***^
		Men	-	-	24.4	37.0	45.2	51.4	57.3	32.9	15.3^***^
		Women	-	-	23.8	40.3	49.1	62.4	62.4	38.6	16.2^***^
	Treatment										
		Total	-	-	17.3	26.9	37.3	45.5	50.3	33.0	22.0^***^
		Men	-	-	17.5	24.6	35.8	39.8	48.8	31.3	22.4^***^
		Women	-	-	17.1	28.6	38.4	49.9	51.5	34.4	21.3^***^
	Control										
		Total	-	-	62.3	73.5	78.8	84.3	84.0	21.7	4.0^***^
		Men	-	-	63.1	80.6	79.3	88.4	85.7	22.6	2.7
		Women	-	-	61.8	68.8	78.5	81.8	82.7	20.9	4.5^***^

Values are presented as weighted %; all estimates are crude.

1 The annual percent change (APC) is significantly different from 0.

*** p<0.001.

**Table 5.** Trends in the prevalence of diabetes among Koreans aged ≥30 years^1^

Variables			2005	2007	2008	2009	2010	2011	2012	2013	2014	2015	2016	2017	2018	Difference (2005-2018)	APC^2^ 2005-2018	APC and year of any significant change in trend slope		
																		Before	Change year	After
Total (≥30 yr)			9.1	9.5	9.7	9.6	9.6	9.7	9.0	11.0	10.1	9.5	11.3	10.4	10.4	1.3	1.2^***^	1.4^***^	2016	-1.0
Men																				
	Age (yr)																			
		Total	10.5	11.8	10.6	10.7	11.0	11.9	10.1	12.8	12.5	11.0	12.9	12.4	12.9	2.4	1.6^***^	0.4	2009	1.9^***^
		30-39	1.7	6.7	2.5	2.8	3.3	3.7	1.7	3.7	2.0	2.8	3.6	3.0	2.7	1.0	-1.0	7.1	2008	-1.8
		40-49	9.3	6.9	10.2	6.9	8.4	8.5	4.4	10.2	10.0	9.5	9.8	8.4	11.7	2.4	1.4	-2.6	2012	6.0
		50-59	18.9	16.8	15.2	15.7	17.7	19.5	15.6	16.3	15.8	12.6	17.4	19.1	16.2	-2.7	-0.2	-1.5	2015	6.0
		60-69	17.9	26.1	21.2	25.2	16.0	23.4	25.6	28.5	30.0	22.4	25.5	23.1	24.6	6.7	1.6	4.0	2014	-3.7
		≥70	16.7	14.4	15.1	18.9	24.5	18.2	22.8	22.5	24.4	23.5	26.4	27.7	27.7	11.0	4.8^***^	8.1^***^	2010	3.9^***^
	Household income^3^																			
		Low	9.8	10.3	15.3	12.5	13.9	12.5	9.7	15.3	17.0	14.0	16.0	17.7	15.3	5.5	3.2^***^	9.8	2008	2.5
		Low-middle	10.0	9.1	9.4	10.3	11.9	10.6	11.3	14.7	12.4	15.5	12.1	11.2	16.3	6.3	3.3^***^	4.2^***^	2015	-0.3
		Middle	11.1	13.2	10.5	12.0	8.4	12.7	7.6	10.1	9.6	8.4	12.9	13.2	10.6	-0.5	0.6	-2.1	2014	7.9
		Middle-high	9.9	15.5	7.6	10.2	8.9	9.3	9.5	13.2	10.8	7.0	11.8	11.2	11.6	1.7	0.7	-3.0	2010	2.4
		High	12.2	11.1	10.9	8.0	11.0	14.0	12.3	11.2	13.4	10.7	11.0	10.1	10.9	-1.3	-0.2	1.0	2014	-3.3
Women																				
	Age (yr)																			
		Total	7.6	7.2	8.5	8.4	8.2	7.5	8.0	9.0	7.9	8.0	9.6	8.4	7.9	0.3	0.5	1.1	2016	-4.5
		30-39	1.2	1.5	1.7	2.3	2.3	1.3	2.0	1.2	2.4	3.0	1.8	0.9	2.2	1.0	1.6	16.0	2009	-1.2
		40-49	5.3	4.4	4.9	4.7	4.8	4.6	5.8	4.4	4.9	4.5	6.1	5.0	3.3	-2.0	-0.9	0.9	2016	-17.5
		50-59	9.1	9.9	9.9	8.9	8.6	8.2	9.5	9.0	8.1	6.9	11.0	11.0	9.8	0.7	0.8	-1.3	2014	6.2
		60-69	18.3	14.5	21.1	19.2	19.1	16.0	15.5	22.1	16.1	17.1	18.3	16.2	16.3	-2.0	-1.2	3.2	2008	-1.7
		≥70	18.5	19.3	21.9	23.8	22.7	23.7	21.5	31.3	23.6	25.0	30.9	28.1	25.7	7.2	2.8^***^	3.8^***^	2016	-4.4
	Household income^3^																			
		Low	6.1	10.5	11.0	8.7	8.7	8.0	10.9	11.8	11.6	11.4	11.8	11.5	10.6	4.5	2.8^***^	14.6	2008	1.7
		Low-middle	8.2	9.8	9.4	8.4	10.4	7.5	11.8	8.7	8.0	8.9	9.7	9.3	9.6	1.4	0.4	3.2	2008	0.1
		Middle	7.7	7.0	7.5	8.5	8.6	7.8	5.6	7.9	5.2	6.8	10.8	7.7	7.7	0.0	0.5	-1.6	2014	6.2
		Middle-high	8.8	3.3	7.1	7.3	6.5	7.9	6.9	8.6	7.1	5.7	6.6	7.1	6.4	-2.4	-1.2	-5.1	2008	-0.6
		High	7.4	5.3	6.2	8.9	7.2	6.5	3.4	8.2	7.6	7.6	8.5	6.7	5.2	-2.2	-0.1	1.7	2016	-17.5

Values are presented as weighted %.

1 Age-standardized prevalence was calculated using the 2005 Population Projections for Korea.

2 The annual percent change (APC) is significantly different from 0.

3 Calculated as monthly household income divided by square root of the number of persons in the household, categorized into quantiles according to age and gender.

*** p<0.001.

**Table 6.** Trends in the prevalence of hypercholesterolemia among Koreans aged ≥30 years^1^

Variables			2005	2007	2008	2009	2010	2011	2012	2013	2014	2015	2016	2017	2018	Difference (2005-2018)	APC^2^ 2005-2018	APC and year of any significant change in trend slope		
																		Before	Change year	After
Total (≥30 yr)			8.0	10.7	10.8	11.4	13.4	13.8	14.4	14.9	14.6	17.9	19.9	21.5	21.4	13.4	7.6^***^	10.4	2008	7.3^***^
Men																				
	Age (yr)																			
		Total	7.3	9.3	9.5	10.8	13.0	12.6	12.2	13.6	13.9	16.4	19.3	20.0	20.9	13.6	8.2^***^	7.4^***^	2014	9.9^***^
		30-39	5.9	7.5	7.5	8.4	8.9	10.3	11.3	11.9	7.6	10.6	10.8	14.8	15.1	9.2	6.4^***^	5.4^***^	2016	15.0
		40-49	8.7	11.0	10.5	11.0	14.3	10.4	8.1	12.1	16.3	18.6	20.2	18.3	22.9	14.2	7.7^***^	2.7	2012	12.4^***^
		50-59	7.9	10.9	10.9	14.0	18.7	16.2	16.0	13.5	18.3	16.2	28.1	24.8	20.8	12.9	7.3^***^	15.0	2010	5.5^***^
		60-69	8.8	9.6	10.5	12.8	13.3	17.0	16.6	18.8	20.3	23.2	27.2	25.8	27.0	18.2	9.4^***^	10.9^***^	2016	1.3
		≥70	3.6	6.6	8.4	8.5	10.6	13.5	14.5	16.8	9.7	20.1	16.4	25.3	25.7	22.1	12.5^***^	32.8	2008	11.7^***^
	Household income^3^																			
		Low	8.6	8.4	12.5	10.8	14.1	13.2	10.8	13.2	10.9	15.3	21.0	23.7	18.9	10.3	7.1^***^	4.2	2014	12.7
		Low-Middle	6.3	6.5	6.9	8.6	11.8	12.3	14.7	10.6	12.9	16.2	16.3	16.9	21.2	14.9	9.1^***^	13.1^***^	2011	7.4^***^
		Middle	7.7	11.5	9.4	12.7	13.2	13.6	9.7	14.1	15.1	15.4	18.8	19.6	23.1	15.4	7.4^***^	5.7^***^	2015	13.2
		Middle-high	6.5	8.0	7.8	9.8	10.8	10.0	11.5	17.1	13.6	16.2	20.3	17.2	20.9	14.4	9.5^***^	10.3^***^	2016	3.0
		High	6.8	11.7	10.2	11.9	13.2	13.9	15.8	13.5	17.4	17.5	19.5	23.3	20.4	13.6	8.0^***^	13.5^***^	2009	7.1^***^
Women																				
	Age (yr)																			
		Total	8.4	11.5	11.8	11.8	13.4	14.8	16.3	15.9	14.9	19.1	20.2	22.6	21.4	13.0	7.1^***^	11.3	2008	6.8^***^
		30-39	1.8	3.5	4.3	4.1	3.1	5.0	5.7	4.9	4.4	5.8	8.0	7.7	9.2	7.4	9.8^***^	26.0	2008	8.5^***^
		40-49	5.5	5.7	6.5	7.7	6.3	9.6	10.4	8.0	9.1	12.6	9.6	17.9	10.5	5.0	7.4^***^	8.0^***^	2016	2.5
		50-59	15.2	19.1	19.5	21.0	23.3	22.4	28.0	26.8	19.6	28.5	33.7	31.7	31.9	16.7	5.5^***^	6.1^***^	2016	0.9
		60-69	17.4	26.5	24.9	22.5	29.5	35.8	31.2	34.5	32.8	40.6	47.3	41.4	44.3	26.9	6.6^***^	7.8^***^	2016	-1.5
		≥70	13.2	20.5	20.2	17.6	27.5	19.8	26.4	29.0	34.0	34.4	29.0	42.6	42.9	29.7	8.6^***^	8.0^***^	2016	11.9
	Household income^3^																			
		Low	8.4	13.5	10.9	12.4	15.5	15.3	15.2	16.3	16.4	20.4	21.1	22.7	21.3	12.9	7.0^***^	11.1^***^	2010	5.9^***^
		Low-middle	8.0	10.2	10.5	12.9	11.9	13.6	17.5	15.5	15.3	17.5	17.9	25.2	24.8	16.8	8.5^***^	7.3^***^	2016	16.7
		Middle	8.0	11.8	10.8	11.4	11.0	13.5	14.6	15.9	15.7	19.9	20.1	22.1	21.3	13.3	7.9^***^	8.6^***^	2016	3.4
		Middle-high	9.4	8.8	13.0	11.2	12.4	16.2	18.7	14.3	13.7	18.9	19.6	20.3	19.6	10.2	6.0^***^	8.9	2011	4.6
		High	8.6	13.4	11.5	11.3	16.3	15.9	15.8	17.6	13.8	18.3	22.2	23.2	19.5	10.9	6.3^***^	7.5^***^	2016	-1.5

Values are presented as weighted %

1 Age-standardized prevalence was calculated using the 2005 Population Projections for Korea.

2 The annual percent change (APC) is significantly different from 0.

3 Calculated as monthly household income divided by square root of the number of persons in the household, categorized into quantiles according to age and gender.

*** p<0.001.

## SUPPLEMENTARY MATERIALS

Korean version is available at http://www.e-epih.org/.
