# Supplementary material for: Trends in the prevalence and management of major metabolic risk factors for chronic disease over 20 years: findings from the 1998-2018 Korea National Health and Nutrition Examination Survey
Source: Epidemiol Health. 2021 Apr 19;43:e2021028. doi: 10.4178/epih.e2021028 (PMC8289481; doi:10.4178/epih.e2021028)
Supplement: Supplementary file 1 [file epih-43-e2021028-suppl.docx]

**Title:**

(Korean) 국민건강영양조사 20년(1998-2018): 만성질환 유병률 및 관리수준 추이

(English) Trends in the prevalence and management of major metabolic risk factors for chronic disease over 20 years: findings from the 1998–2018 Korea National Health and Nutrition Examination Survey

**Running title**: Twenty-year trends in the prevalence of chronic disease

**Authors:**

김윤정^1^, 노선진^1^, 우경지^1^, 김혜진^1^, 박수연^1^, 김영택^2^, 박옥^1^, 오경원^1^

Yoonjung Kim^1^, Sun Jin Nho^1^, Gyeongji Woo^1^, Hyejin Kim^1^, Suyeon Park^1^, Youngtaek Kim^2^, Ok Park^1^, Kyungwon Oh^1^

**Affiliations:**

^1^질병관리청 만성질환관리국 건강영양조사분석과, ^2^충남대학교 병원

^1^Division of Health and Nutrition Survey and Analysis, Bureau of Chronic Disease Prevention and Control, Korea Disease Control and Prevention Agency, Cheongju, Korea; ^2^Public Health Medical Service Office, Chungnam National University Hospital, Daejeon, Korea

**Correspondence:** Kyungwon Oh

Division of Health and Nutrition Survey and Analysis, Bureau of Chronic Disease Prevention and Control, Korea Disease Control and Prevention Agency, 187 Osongsaengmyeong 2-ro, Heungduk-gu, Cheongju 28159, Korea

E-mail: [kwoh27@korea.kr](mailto:kwoh27@korea.kr)

**ABSTRACT**

**목적:** 국민건강영양조사 자료를 이용하여 우리나라 성인의 비만, 고혈압, 당뇨병, 고콜레스테롤혈증 유병률 및 관리수준의 20년간 변화를 확인하고자 한다.

**방법:** 분석대상은 국민건강영양조사 제1기(1998)~제7기(2016-2018) 검진과 설문조사에 참여한 만30세이상 79,753명이었다. 만성질환 유병률 및 관리수준, 연간 변화율(Annual percent change; APC)을 SAS, Joinpoint 프로그램을 이용하여 산출하였다.

**결과:** 비만 유병률은 20년동안 남자에서 1998년 26.8%에서 2018년 44.7%로 크게 증가한반면(APC=1.9, p<0.001), 여자는 1998년 30.5%에서 2018년 28.3%로 소폭 감소하였다(APC=-0.5, p<0.001). 고혈압 유병률은 남자는 큰 변화 없이 2018년 33.2%이며, 여자는 소폭 감소하여 23.1%이었고(APC=-0.9, p<0.001), 당뇨병 유병률은 남자는 2005년 10.5%에서 2018년 12.9%로 소폭 증가한 반면(APC=1.6, p<0.001), 여자는 큰 변화 없이 8% 수준이었다. 고콜레스테롤혈증 유병률은 남녀 모두 2005년대비 2018년에 3배정도 증가하여 남자는 20.9%(APC=8.2, p<0.001), 여자는 21.4%이었다(APC=7.1, p<0.001). 고혈압과 고콜레스테롤혈증의 인지율, 치료율, 조절률 모두 2∼3배 증가하였고, 당뇨병의 경우 치료율은 증가한 반면 조절률은 변화가 없었다.

**결론:** 지난 20년 동안 비만(남자), 당뇨병, 고콜레스테롤혈증 만성질환의 유병 수준은 악화되고 있는 반면, 만성질환의 인지율, 치료율, 치료자 중 조절률 등 관리지표는 지속적으로 개선되고 있었다.

**주제어**: 국민건강영양조사, 비만, 고혈압, 당뇨병, 고콜레스테롤혈증

**INTRODUCTION**

비감염성질환(noncommunicable diseases; NCDs)로 알려진 만성질환은 심뇌혈관질환, 암, 만성호흡기질환, 당뇨병이며 유전, 생활습관 등으로 인해 발병하는 질환이다 [1]. NCDs로 인해 2016년에 전세계 사망의 70%에 해당되는 41백만명이 사망하고 사망원인 대부분이 심뇌혈관질환(17.9백만명 사망)이며, 암(9백만명 사망), 만성호흡기질환(3.8백만명 사망), 당뇨병(1.6백만명 사망) 순으로 기여하고 있다 [2]. 세계보건기구는 2013년에 ‘비감염성 질환에 대한 글로벌 액션플랜 2013-2020’을 수립하고, 만성질환으로 인한 조기사망률 25% 감소를 목표로 설정하고, 국가별로 관련 정책 수립 및 성과지표 관리를 촉구하고 있다 [3].

우리나라의 경우도 전체 사망원인의 69.1%를 차지하는 10대 사망원인은 암, 심장질환, 폐렴, 뇌혈관질환, 고의적 자해(자살), 당뇨병, 간질환, 만성하기도 질환, 알츠하이머병, 고혈압성질환으로 대부분 만성질환이 기여하고 있다 [4]. 이를 고려하여 국민건강증진종합계획 2020 (Health Plan 2020; HP2020)에 질병부담이 높은 4개의 만성질환(심뇌혈관질환, 당뇨병, 만성호흡기질환, 암)의 집중 관리를 위해 선행단계인 고혈압, 당뇨병, 고콜레스테롤혈증, 비만 유병률을 낮추고, 관리수준을 개선하기 위한 목표를 수립하였고, 국민건강영양조사를 통해 목표치 대비 성과를 모니터링하고 있다 [5].

본 논문에서는 국민건강영양조사(1998년~2018년) 자료를 이용하여 우리나라 성인의 비만, 고혈압, 당뇨병, 고콜레스테롤혈증 유병률 및 관리수준의 20년간 변화를 확인하고자 한다.

**MATERIALS AND METHHODS**

**조사대상**

국민건강영양조사는 국민의 건강수준, 건강행태, 식품 및 영양섭취 실태에 대한 국가 단위의 통계를 산출하기 위한 목적으로 실시하고 있다 [6]. 조사 표본은 2단계 층화집락표본추출방법을 적용하여 조사구 및 가구를 1, 2차로 추출하며, 연간 약 200개 조사구, 조사구당 20~23개 가구이다. 조사대상은 표본가구 내 만1세 이상의 모든 가구원이다.

본 연구의 분석대상은 국민건강영양조사 제1기(1998)부터 제7기(2016-2018) 검진조사와 설문조사를 모두 참여한 만30세 이상이며, 고콜레스테롤혈증 분석 시 공복시간이 8시간 미만인 경우(4,343명), 비만 분석 시 임산부(303명)를 분석대상에서 제외하였다.

**검진조사**

국민건강영양조사 검진조사는 제1기(1998), 제2기(2001), 제3기(2005), 제4기 1차년도(2007)에는 조사지역과 인접한 공간에 설치한 센터에서 수행하였다. 2008년 검진차량이 도입됨에 따라 이후 검진조사는 이동검진차량에서 질병관리청(이전 질병관리본부) 소속의 전문조사원에 의해 수행되었다. 신장은 이동검진차량 벽에 부착된 신장계 (SECA 225; Seca GmbH, Hamburg, Germany)를 이용하여 0.1cm 단위로 측정하였고, 체중은 이동형 체중계를 이용하여 0.1kg 단위로 측정하였다. 혈압은 수은혈압계 (Baumanometer; Baum, Copiague, NY, USA)을 이용하여 5분 안정 후 앉은 자세에서 조사 대상자의 오른팔에서 혈압을 측정하였다(1998년과 2001년 2회 측정한 평균, 2005년이후 3회 측정하고, 2회와 3회 측정값의 평균을 최종 혈압 측정치로 사용). 혈압측정 및 신체계측은 관련 학회 또는 전문기관에서 질관리를 실시하고 있다 [7, 8].

당뇨병 및 이상지질혈증 유병률 산출을 위해 대상자의 혈액을 수집하였고, 최소 8시간 공복상태를 유지한 대상자의 자료만 분석에 사용하였다. 혈액 채취 후 8mL SST 용기는 상온에 30분간 수직으로 세워 둔 후 3000rpm으로 15분간 원심분리하여 냉장고에 보관하였고, 3ml EDTA 용기는 항응고제와 혈액이 잘 혼합되어 응고되지 않도록 roller mixer에서 10분간 혼합 후 냉장 보관한 후 2–8℃ 상태로 진단검사 분석기관에 당일 배송되어 분석하였다.

진단의학검사 분석기관이 2005년, 2008년에 변경되면서 혈당 및 콜레스테롤 분석 장비 및 시약이 변경되었으며, 채혈용기는 2014년부터 NaF에서 SST 용기로 변경되었다 [9]. 2008년부터 총콜레스테롤과 공복혈당은 Enzymatic method (Hitachi Automatic Analyzer 7600; Hitachi, Tokyo, Japan), 당화혈색소는 High Performance Liquid Chromatography (Tosoh G8; Tosoh, Tokyo, Japan) 방법으로 분석하였다. 진단의학검사 전 과정은 2005년부터 진단의학검사학회와 공동으로 질관리를 실시하고 있다 [10]. 만성질환 유병률 및 관리지표 산출에 필요한 평생 의사진단 경험 및 현재 약복용 여부는 면접방법으로 조사하였다.

**만성질환 유병 및 관리 지표 정의**

신장과 체중을 이용하여 체질량지수(Body mass index; BMI)를 산출하였고, 대한비만학회 기준에 근거하여 BMI ≥25 kg/m^2^를 비만으로 분류하였다 [11]. 고혈압은 대한고혈압학회 기준에 따라 수축기 혈압 140mmHg 이상이거나 이완기 혈압이 90mmHg 이상 또는 고혈압 약물을 복용하는 경우로 정의하였다 [12]. 당뇨병은 대한당뇨병학회 기준에 따라 공복혈당이 126mg/dL 이상이거나 의사진단을 받았거나 혈당강하제 복용 또는 인슐린 주사를 사용하는 경우로 정의하였다 [13]. 고콜레스테롤혈증은 한국지질·동맥경화학회 기준에 따라 혈중 총콜레스테롤이 240mg/dL이상이거나 콜레스테롤강하제를 복용하는 경우로 정의하였다 [14].

당뇨병과 고콜레스테롤혈증 유병률과 관리지표는 1998년과 2001년 조사의 경우 채혈전 금식 시간, 당화혈색소 측정 정보에 대한 재확인이 어렵고, 고콜레스테롤혈증 약복용 여부 에 관한 조사항목이 없어 유병률 산출에 필요한 조사항목을 모두 포함하고 전문기관에서 진단의학검사 질관리를 실시한 2005년부터 산출하였다.

고혈압, 당뇨병, 고콜레스테롤혈증 인지율은 유병자 중에 의사로부터 진단 받은 경험이 있다고 한 사람의 분율이며, 치료율은 유병자 중 현재 약복용을 하는 사람의 분율이다. 조절률은 치료자 중 치료 목표(고혈압: 수축기 혈압 < 140 mm Hg, 이완기 혈압 < 90mmHg, 당뇨병: 당화혈색소 < 6.5%, 고콜레스테롤혈증: 혈중 콜레스테롤 < 200 mg/dL)에 도달한 사람의 분율로 정의하였다.

**통계분석방법**

자료 분석은 SAS version 9.4 (SAS Institute Inc., Cary, NC, USA)와 Joinpoint Regression Program version 4.1.1.1 (US National Cancer Institute, Bethesda, MD, USA)를 이용하였다. 모든 결과는 목표 모집단인 대한민국에 거주하는 국민의 특성을 잘 대표할 수 있도록 가중치를 적용하여 복합표본설계분석방법으로 산출하였다.

비만, 고혈압, 당뇨병, 고콜레스테롤혈증 유병률의 성별과 소득수준별 추이는 연령구조 차이에 따른 영향을 보정하기 위해 2005년 추계인구로 표준화하였고, 연령표준화율은 SAS(PROC SURVEYREG)를 이용하여 계산하였다. 연령별 유병률과 관리지표는 조율값이며, SAS(PROC SURVEYMEANS)를 이용하여 계산하였다. 관리지표인 인지율, 치료율, 조절률은 당해연도 자료만으로 성별, 연령별 일부 구간에서 유병자 수가 불충분하여 상대표준오차가 25%이상이 존재하고 연령대별 자료수가 20명 미만인 경우가 있어 3년 자료를 통합하였고, 연령표준화율 산출이 불가하여 조율을 제시하였다. SAS를 이용하여 산출된 연령표준화 유병률 및 표준오차를 Joinpoint 프로그램에 적용하여 변곡점(joinpoint)을 0, 1개로 설정하여 모형을 추정하였고, 연간변화율(Annual Percent Change; APC)을 산출하였다. APC는 유의수준 0.05하에서 연간변화율이 “0”인지 검증하였고, 최적 모형에 대한 통계적 유의성 검정에는 Joinpoint Regression Program의 Monte Carlo method를 사용하였다.

**윤리 성명**

본 연구는 질병관리청 연구윤리심의위원회를 통해서 연도별 계획에 따라 승인을 받았다 (2007-2014년, 2018년). 일부 연도에는 생명윤리법 제2조 제1호 및 동 시행규칙 제2조 제2항 제1호에 따라 심의면제를 받았다 (2015-2017년).

**RESULTS**

**대상자 특성**

국민건강영양조사 제1기(1998)-제7기(2016-2018) 검진조사 대상자의 일반적 특성은 Table 1에 제시하였다. 조사 대상자 수는 만30세이상 79,753명이었고, 연령별로는 1998년에서 2018년 동안 20-40대는 감소, 50대 이상은 증가하였다.

**비만**

비만 유병률(연령표준화, ≥만30세)은 남자의 경우 1998년 26.8%에서 2018년 44.7%로 크게 증가한 반면(APC=1.9, p<0.001), 여자는 같은 기간 30.5%에서 28.3%로 소폭 감소하였다(APC=-0.5, p<0.001). 20년동안 특히 2005년 이후 남자는 30대와 60대 이상에서 유의하게 증가한 반면 여자는 50대와 60대에서 뚜렷하게 감소하였다(Table 2). 소득수준에 따른 비만 유병률 추이의 경우 남자는 모든 그룹에서 증가한 반면, 여자는 소득수준이 중 그룹 이상에서 감소하였으며 특히 상 그룹은 2007년 이후 큰 폭으로 감소하였다(APC=-2.3, p<0.001). 여자의 경우 소득수준 상-하 그룹의 비만 유병률 차이가 1998년 -2.1%p(28.0%, 30.1%)에서 2018년 -17.5%p(18.0%, 35.5%)로 약8배 커졌다(Table 2).

**고혈압**

고혈압 유병률(연령표준화, ≥만30세)은 남자는 1998년 32.4%에서 2018년 33.2%로 20년 동안 큰 변화가 없었으나 여자는 1998년 26.8%에서 2018년 23.1%로 소폭 감소하였다(APC=-0.9, p<0.001). 연령별 유병률 추이의 경우 남자는 30, 40대가 각각 2009년, 2007년 이후 증가하였고(APC=3.5, 1.8, p<0.001), 여자는 50, 60대가 각각 2005년, 2011년 이후 감소하였으며(APC=-1.8, -2.5, p<0.001) 남녀 모두 70대 이상에서 2005년 이후 증가하였다 (APC=2.1, 1.1, p<0.001) (Table 3). 소득수준에 따른 유병률의 경우 남자는 모든 그룹에서 20년 동안 유의한 변화가 없었으나 여자는 중 이상 그룹에서 2016년 이전까지 유의하게 감소하였다. 소득수준 상-하 그룹의 유병률 차이는 20년 동안 남녀 모두 커졌으며, 특히 여자의 경우 1998년 -2.7%p (25.1%, 27.8%)에서 2018년 –8.5%p (17.4%, 25.9%)로 약 3배 커졌다.

고혈압 인지율은 1998년 23.5%에서 2016-2018년 69.1%, 치료율은 1998년 20.4%에서 2016-2018년 65.3%, 치료자 중 조절률은 1998년 23.8%에서 2016-2018년 73.1%로 각각 큰 폭으로 증가하여(APC=9.2, 11.1, 6.2, p<0.001) (Table 4), 고혈압을 인지하면 대부분 치료를 하고, 치료하면 조절되고 있었다. 연령별로는 30대와 40대의 인지율(2016-2018년 19.8%, 44.8%)과 치료율(2016-2018년 16.9%, 38.2%)이 다른 연령대에 비해 낮았고 특히 30대가 20% 미만으로 매우 낮은 수준이었다 [15].

**당뇨병**

당뇨병 유병률(연령표준화, ≥만30세)은 2005년에서 2018년 동안 남자는 10.5%에서 12.9%로 증가하였고(APC=1.6, p<0.001), 2009년 이후 이 경향이 뚜렷하였다(APC=1.9, p<0.001). 여자는 큰 변화 없이 8% 수준이었다(APC=0.5). 연령별 유병률 추이의 경우 70대이상에서 남자는 2010년 이전에 증가 폭이 컸으며(APC=8.1, p<0.001) 여자는 2016년 이전에 증가 경향이 뚜렷하였다(APC=3.8, p<0.001). 소득수준에 따른 유병률 추이의 경우 남녀 모두 하 그룹에서 증가하였다(APC=3.2, 2.8, p<0.001). 소득수준 상-하 그룹의 유병률 차이는 2005년에서 2018년 동안 남자는 2.4%p(12.2%, 9.8%)에서 -4.4%p(10.9%, 15.3%), 여자는 1.3%p(7.4%, 6.1%)에서 –5.4%p(5.2%, 10.6%)로 소득수준에 따른 차이가 더 커졌다(Table 5).

당뇨병 인지율은 여자에서만 증가하였고(APC=1.6, p<0.001), 치료율은 남녀 모두 증가한 반면(APC=4.7, 5.5, p<0.001), 당뇨병 치료자 중 조절률은 남녀 모두 유의한 변화가 없었다 (Table 4). 연령별로는 30대와 40대의 당뇨병 인지율(2016-2018년 33.6%, 47.9%)과 치료율(2016-2018년 28.5%, 42.4%)이 고혈압과 고콜레스테롤혈증에 비해 높았으나 50% 미만이었다 [15].

**고콜레스테롤혈증**

고콜레스테롤혈증 유병률(연령표준화, ≥만30세)은 2005년에서 2018년 동안 남자는 7.3%에서 20.9%로 13.6%p 증가하였고(APC=8.2, p<0.001), 2014년 이후 크게 증가하였다(APC=9.9, p<0.001). 여자는 8.4%에서 21.4%로 13.0%p로 증가하였고(APC=7.1, p<0.001), 2008년 이후 증가하였다(APC=6.8, p<0.001). 연령별 유병률 추이의 경우 남녀 모두 모든 연령에서 증가하였고(p<0.001), 특히 여자 40대 이상의 모든 연령 그룹에서 2016년 이전의 증가 경향이 뚜렷하였다. 50대이전까지는 남자의 유병률이 여자에 비해 약1.5배 이상 높았으나 50대이후는 여자가 남자보다 약1.5배 이상 높았다. 소득수준에 따른 모든 그룹의 유병률도 20년 동안 유의하게 증가하였다 (Table 6).

고콜레스테롤혈증 인지율은 2005년 24.0%에서 2016-2018년 60.1%(APC=16.1, p<0.001), 치료율은 2005년 17.3%에서 2016-2018년 50.3%(APC=22.0, p<0.001)로 증가하였고, 남녀 모두 증가 경향을 보였다 (Table 4). 치료자 중 조절률은 여자에서만 2005년 61.8%에서 2016-2018년 82.7%로 증가하였다 (APC=4.5, p<0.001). 연령별로는 30대와 40대에서 인지율(2016-2018년 18.0%, 40.3%)과 치료율(2016-2018년 10.6%, 27.9%)이 50% 수준을 미치지 못하였으며, 특히 30대의 치료율은 10% 수준으로 다른 만성질환과 비교 시 가장 낮았다 [15].

**DISCUSSION**

국민건강영양조사 자료를 이용하여 심뇌혈관질환 대사적 위험요인의 20년간 추이 및 관리수준을 분석한 결과, 비만, 당뇨병, 고콜레스테롤혈증 유병률은 증가하였고, 고혈압과 고콜레스테롤혈증 인지율, 치료율, 조절률은 모두 개선되었으며, 당뇨병은 치료율만 개선 되었다 (Table 4).

비만 유병률(연령표준화, ≥만30세)은 20년간 남자의 경우 모든 연령에서 뚜렷하게 증가하였고(1998년 26.8%, 2018년 44.7%, APC=1.9) 여자는 소폭 감소하여(1998년 30.5%, 2018년 28.3%, APC=-0.5) HP2020 목표인 남자 37.0%, 여자 27.0%를 모두 미달성 하였다. 국가마다 비만 기준과 산출 연령이 다르므로 국가 간 비교를 위해 BMI ≥25 kg/m^2^, 20세 이상으로 산출하여 비교하였다. 우리나라의 유병률(연령표준화, ≥19세, 2018년)은 남자 42.8%, 여자 25.5%로, 미국(연령표준화, ≥20세, 2013-2016년)의 남자 74.6%, 여자 67.4%에 비해 2배정도 낮은 수준이었다 [16]. 반면 생활습관이 유사한 일본(연령표준화, ≥20세, 2018년)의 남자 31.6%, 여자 19.6% 보다는 우리나라의 유병률이 더 높은 수준이었다 [17]. 남자의 유병률이 지속적인 증가 경향으로 특히 30대, 40대의 유병률이 50% 수준이고, 20, 30대 여자가 증가 경향인 점이 우려되는 상황이다. 비만이 만성질환의 강력한 위험요인임을 고려한다면 영양, 신체활동, 보건의료 등 다양한 분야가 유기적으로 협력하여 2018년에 수립한 국가비만종합대책의 적극적인 추진이 필요한 시점이라고 볼 수 있다.

고혈압 유병률(연령표준화, ≥만30세)은 20년간 남자는 큰 변화가 없이 2018년 33.2%, 여자는 소폭 감소하여 2018년 23.1%로, HP2020 목표인 23.0%를 달성하지 못했다. 고혈압 유병률을 국가별로 비교 시, 우리나라의 유병률(연령표준화, 2018년, ≥만19세)은 남자 27.6%, 여자 18.3%로 미국(연령표준화, ≥20세, 2015-2016년)의 남자 31.3%, 여자 28.7%에 비해 낮았고, 일본(연령표준화, ≥20세, 2018년)의 남자 28.0%, 여자 19.0%와 유사한 수준이었다 [16, 17]. 고혈압 유병률이 다른 만성질환에 비해 크게 증가하지 않은 것은 주요한 위험요인 [18] 중 비만, 신체활동은 악화되었음에도 불구하고, 1999년 이후 추진된 정책(건강생활실천(1999년), 만성질환관리(2000년), 권역심뇌혈관 질환 센터운영 및 홍보(2005년))에 의해 혈압 관리의 중요성에 관한 인식 및 생활습관 개선과 함께 고혈압을 더 빨리 인지하고 치료가 증가한 점 등이 복합적으로 영향을 미쳤을 것으로 여겨진다 [19-21]. 그러나 맞춤형 방문 건강관리(2007년), 고혈압·당뇨병 등록관리(2007년), 생애주기별 국가건강검진(2007년), 건강검진사후관리 (2008년), 의원급 만성질환관리(2012년), 나트륨저감화(2012년) 등 후속사업이 추진되었음에도 관리지표 개선 정도는 2007년 이후 둔화되었다. 이는 다른 연령대에 비해 30, 40대의 인지율과 치료율이 개선없이 낮은 수준을 유지하는 것과 관련 있다고 여겨지며, 이 연령의 관리지표 개선을 위한 효과적인 방안으로 고혈압에 관한 관심과 주기적인 건강검진 유도가 필요하다고 제안된 바 있다 [22].

당뇨병(공복혈당 기준) 유병률(연령표준화, ≥만30세)은 남자는 2005년 10.5%에서 2018년 12.9%으로 소폭 증가한 반면 여자는 유의한 변화 없이 8% 수준이었으며, HP2020 목표인 9.7%를 달성하지 못했다. 심뇌혈관질환 선행단계인 공복혈당장애는 2018년 남자 33.2%, 여자 23.4%로 당뇨병 유병률보다 2-3배 높았다. 미국과 핀란드의 경우 당뇨병 예방 프로그램을 운영하여 공복혈당장애 또는 내당능장애와 같은 당뇨병 전단계를 위한 생활습관 프로그램을 제공하고 있다 [23, 24]. 우리나라도 당뇨병 전단계의 규모 및 추이를 고려 시 당뇨병 관리 뿐만 아니라 당뇨병으로 이행되지 않도록 당뇨병 전단계를 대상으로 한 예방사업의 적극적인 추진이 필요하다.

고콜레스테롤혈증 유병률(연령표준화, ≥만30세)은 남녀 모두 증가하여 2018년 기준 남자 20.9%, 여자 21.4%였으며, HP2020 목표인 13.5%를 달성하지 못했다. 고콜레스테롤 혈증 유병률이 30-40대에서 남자(2018년 15.1%, 22.9%)가 여자(2018년 9.2%, 10.5%)보다 약2배 높은 이유는 고콜레스테롤혈증의 다양한 위험요인 중 높은 흡연율(2018년, 30대 39.9%, 40대 44.1%)과 관련이 있는 것으로 여겨진다 [15, 25]. 또한 이 연령대의 남자는 다른 연령대에 비해 에너지 및 지방 과잉섭취가 높은 반면(남 10.6% 9.6%; 여 5.8%, 4.7%), 건강식생활 실천이 낮았다(남 36.3%, 37.4%; 여 52.1%, 55.6%) [15]. 50대 이상에서는 여자의 유병률이 남자보다 높았으며 폐경으로 인한 호르몬 변화가 체내 지질대사에 미치는 영향에 의한 것으로 보인다 [26].

성별, 연령별로 만성질환의 유병 현황을 비교 시, 남자는 비만 유병률이 30대에서 가장 높고, 당뇨병 및 고콜레스테롤혈증 유병률이 40대부터 급격하게 증가하였다. 남자 30-40대의 경우 에너지 및 지방 과잉 섭취가 높고(10.6%, 9.6%), 월간폭음이 많고(55.9%, 59.3%), 유산소신체활동실천이 낮은(55.3%, 48.8%) 생활습관이 [15] 40대 이후의 당뇨병 및 고콜레스테롤혈증 유병률 상승에 영향을 미친 것으로 사료된다. 국민건강영양조사 자료 기반의 연령, 기간, 코호트 효과를 분석한 연구에서도 [27] 1970년대 이후 출생 코호트에서 비만, 고콜레스테롤혈증 유병률이 증가하는 경향을 보였다. 1970년 출생 코호트의 경우 2018년 시점에 40대에 해당하므로 이들의 연령이 증가함에 따라 비만, 고콜레스테롤혈증 등 만성질환 유병률 증가가 예상되므로 만성질환 관리 방안 강화가 필요하다고 볼 수 있다.

만성질환의 관리지표는 전반적으로 개선되었으나 당뇨병의 조절률은 큰 변화 없이 25%수준으로 다른 만성질환에 비해 낮았다. 이에 관해 노인 인구의 증가, 이환 기간의 증가, 낮은 건강생활 실천율 및 당뇨병 약제 순응도 등을 관련요인으로 설명하고 있다 [28-30]. 모든 만성질환의 인지율이 30-40대에서 낮아 만성질환을 예방하기 위해서는 젊은 연령층의 인지율을 높이고 생활습관이 고착화되기 전에 올바른 생활습관 형성을 위한 프로그램이 필요함을 시사해 준다. 또한 여자의 상-하 그룹간 소득수준에 따른 만성질환 유병률 차이가 비만 15.4%p(1998년 -2.1%p, 2018년 -17.5%p), 고혈압 5.8%p(1998년 -2.7%p, 2018년 -8.5%p), 당뇨병 6.7%p(2005년 1.3%p, 2018년 -5.4%p)로 큰 폭으로 커져 건강격차를 줄이기 위한 사업의 필요성을 뒷받침해 주고 있다.

국민건강영양조사를 통해 지난 20년동안 비만, 고콜레스테롤혈증의 유병 수준은 악화되고 고혈압, 고콜레스테롤혈증, 당뇨병의 관리지표의 개선 경향이 최근에는 둔화되고 있었다. 향후 만성질환 유병 감소, 건강 격차 감소, 만성질환 관리지표 개선을 목표로 관련 정책의 지속적 추진이 필요하다.

**CONFLICT OF INTEREST**

모든 저자는 본 연구에 대해 표명할 이해상충이 없음

**FUNDING**

없음

**ACKNOWLEDGEMENTS**

지난 20년동안 국민건강영양조사에 참여해주신 대상자께 감사드립니다. 또한, 조사를 전담하여 수행한 조사수행팀, 조사 지원과 자문을 주신 관련학회 및 전문가 자문단에게도 감사드립니다.

**AUTHOR CONTRIBUTIONS**

Conceptualization: YK, KO. Data curation: SJN, GW. Formal analysis: HK, SP. Funding acquisition: None. Project administra­tion: YK, OP, KO. Visualization: YK, SP. Writing-original draft: YK, YK, OP, KO. Writing-review and editing: YK, KO.

**ORCID**

Yoonjung Kim: *https://orcid.org/0000-0002-8418-0074*; Sun Jin Nho: *https://orcid.org/0000-0001-9553-7940*; Gyeongji Woo: *https:// orcid.org/0000-0001-7741-6539*; Hyejin Kim: *https://orcid.org/ 0000-0001-8859-2095*; Suyeon Park: *https://orcid.org/0000-0001- 8134-8436*; Youngtaek Kim: *https://orcid.org/0000-0003-0139-7620*; Ok Park: *https://orcid.org/0000-0002-9477-9523*; Kyungwon Oh: *https://orcid.org/0000-0001-8097-6078*

**REFERENCES**

1. World Health Organization. Noncommunicable diseases [cited 2020 Sep 21]. Available from: https://www.who.int/news-room/ fact-sheets/detail/noncommunicable-diseases.

2. World Health Organization. Global health estimates 2016: deaths by cause, age, sex, by country and by region, 2000-2016; 2018 [cited 2020 Sep 21]. Available from: http://www.who.int/health­info/global_burden_disease/estimates/en/.

3. World Health Organization. Global status report on noncommu­nicable diseases 2014 [cited 2020 Sep 21]. Available from: https:// www.who.int/nmh/publications/ncd-status-report-2014/en/.

4. Statistics Korea. Annual report on the causes of death statistics; 2019 [cited 2020 Sep 22]. Available from: https:// kostat.go.kr/portal/korea/kor_nw/1/1/index.board (Korean).

5. Ministry of Health and Welfare of Korea. The National Health Plan 2020; 2015 [cited 2020 Sep 21] Available from: http://www. mohw.go.kr/react/jb/sjb030301vw.jsp?PAR_MENU_ID=03& MENU_ID=0319&CONT_SEQ=330479 (Korean).

6. Kweon S, Kim Y, Jang MJ, Kim Y, Kim K, Choi S, et al. Data re­source profile: the Korea National Health and Nutrition Exami­nation Survey (KNHANES). Int J Epidemiol 2014;43:69-77.

7. Korea Centers for Disease Control and Prevention. Quality con­trol and assurance of blood pressure measurement for the Korea National Health and Nutrition Examination Survey (2018, 7th). Cheongju: Korea Centers for Disease Control and Prevention; 2018, p. 17-216 (Korean).

8. Korea Centers for Disease Control and Prevention. Quality con­trol of anthropometric measurement (height, weight, waist cir­cumference) for the Korea National Health and Nutrition Exami­nation Survey (2018, 7th). Cheongju: Korea Centers for Disease Control and Prevention; 2018, p. 26-145 (Korean).

9. Lee YW, Cha YJ, Chae SL, Song J, Yun YM, Park HI, et al. Effec­tiveness of sodium fluoride as a glycolysis inhibitor on blood glu­cose measurement: comparison of blood glucose using specimens from the Korea National Health and Nutrition Examination Sur­vey. Korean J Lab Med 2009;29:524-528 (Korean).

10. Korea Centers for Disease Control and Prevention. Quality con­trol of the clinical laboratory for the Korea National Health and Nutrition Examination Survey (2018, 7th). Cheongju: Korea Centers for Disease Control and Prevention; 2018, p. 23-180 (Korean).

11. Korean Society for the Study of Obesity. Guideline for the man­agement of obesity in Korea; 2018 [cited 2020 Sep 21]. Available from: http://general.kosso.or.kr/html/?pmode=BBBS000130000 3&page=1&smode=view&seq=1358&searchValue=&searchTi tle=strTitle (Korean).

12. Korean Society of Hypertension. Guideline for the management of hypertension; 2018 [cited 2020 Sep 21]. Available from: http:// www.koreanhypertension.org/reference/guide?mode=read& idno=4246 (Korean).

13. Korean Diabetes Associations. Treatment guideline for diabetes in Korea 6th ed; 2019 [cited 2020 Sep 21]. Available from: https:// www.diabetes.or.kr/pro/publish/guide.php?code=guide&mode= list&year_v=2019 (Korean).

14. Korean Society of Lipid and Atherosclerosis. Korean guidelines for the management of dyslipidemia 4th ed; 2018 [cited 2020 Sep 21]. Available from: [https://www.lipid.or.kr/bbs/index. html?code=care&category=&gubun=&page](https://www.lipid.or.kr/bbs/index.%20html?code=care&category=&gubun=&page)=1&number=957&mode=view&keyfield=&key= (Korean).

15. Korea Centers for Disease Control and Prevention. Korea health statistics 2018: Korea National Health and Nutrition Examination Survey (KNHANES VII-3); 2019 [cited 2020 Sep 21]. Available from: https://knhanes.kdca.go.kr/knhanes/sub04/sub04_04_01. do (Korean).

16. National Center for Health Statistics. Health, United States, 2018; 2019 [cited 2020 Sep 21]. Available from: https://www.cdc.gov/ nchs/data/hus/hus18.pdf.

17. National Institute of Health and Nutrition. 2018 National Health and Nutrition Survey Japan; 2019 [cited 2020 Sep 21]. Available from: https://www.nibiohn.go.jp/eiken/kenkounippon21/down­load_files/eiyouchousa/2018.pdf.

18. Carey RM, Muntner P, Bosworth HB, Whelton PK. Reprint of: prevention and control of hypertension: JACC Health Promotion Series. J Am Coll Cardiol 2018;72:2996-3011.

19. Park HK, Lee Y, Kang BW, Kwon KI, Kim JW, Kwon OS, et al. Progress on sodium reduction in South Korea. BMJ Glob Health 2020;5:e002028.

20. NCD Risk Factor Collaboration (NCD-RisC). Long-term and recent trends in hypertension awareness, treatment, and control in 12 high-income countries: an analysis of 123 nationally repre­sentative surveys. Lancet 2019;394:639-651.

21. Kang SH, Kim SH, Cho JH, Yoon CH, Hwang SS, Lee HY, et al. Prevalence, awareness, treatment, and control of hypertension in Korea. Sci Rep 2019;9:10970.

22. Jeon YW, Kim HC. Factors associated with awareness, treatment, and control rate of hypertension among Korean young adults aged 30-49 years. Korean Circ J 2020;50:1077-1091.

23. Centers for Disease Control and Prevention. National diabetes prevention program; 2020 [cited 2020 Sep 21]. Available from: https://www.cdc.gov/diabetes/prevention/index.html.

24. Uusitupa M, Louheranta A, Lindström J, Valle T, Sundvall J, Eriks­son J, et al. The Finnish diabetes prevention study. Br J Nutr 2000; 83 Suppl 1:S137-S142.

25. Jellinger PS, Handelsman Y, Rosenblit PD, Bloomgarden ZT, Fon­seca VA, Garber AJ, et al. American Association of Clinical En­docrinologists and American College of Endocrinology Guide­lines for management of dyslipidemia and prevention of cardio­vascular disease. Endocr Pract 2017;23(Suppl 2):1-87.

26. Park JH, Lee MH, Shim JS, Choi DP, Song BM, Lee SW, et al. Ef­fects of age, sex, and menopausal status on blood cholesterol pro­file in the Korean population. Korean Circ J 2015;45:141-148.

27. Korea Centers for Disease Control and Prevention. Trend analysis and association study of chronic diseases and risk factors: findings from the KNHANES 1998-2018. Cheongju: Korea Centers for Disease Control and Prevention; 2020, p. 65-92 (Korean).

28. Shin JY. Trends in the prevalence and management of diabetes in Korea: 2007-2017. Epidemiol Health 2019;41:e2019029.

29. Korea Centers for Disease Control and Prevention. Korea non-communicable disease forum. Cheongju: Korea Centers for Disease Control and Prevention; 2018, p. 9-10 (Korean).

30. Oh SW, Lee HJ, Chin HJ, Hwang JI. Adherence to clinical practice guidelines and outcomes in diabetic patients. Int J Qual Health Care 2011;23:413-419.

**Table 1. Characteristics of health examination participants of Korea National Health and Nutrition Examination Survey**

| Characteristics | | 1998 | | 2001 | | 2005 | | 2007–2009 | | 2010–2012 | | 2013–2015 | | 2016–2018 | |
| --- | --- | --- | --- | --- | --- | --- | --- | --- | --- | --- | --- | --- | --- | --- | --- |
| Total (≥30 yr) | | 6,469 | | 5,500 | | 4,818 | | 15,126 | | 16,431 | | 15,042 | | 16,367 | |
| Men | | 2,938 | (45.4) | 2,410 | (43.8) | 2,065 | (42.9) | 6,389 | (42.2) | 7,033 | (42.8) | 6,410 | (42.6) | 7,121 | (43.5) |
| Age (yr) | |  |  |  |  |  |  |  |  |  |  |  |  |  |  |
|  | 30–39 | 1,968 | (30.4) | 1,667 | (30.3) | 1,226 | (25.4) | 3,498 | (23.1) | 3,403 | (20.7) | 2,758 | (18.3) | 2,920 | (17.8) |
|  | 40–49 | 1,630 | (25.2) | 1,525 | (27.7) | 1,346 | (27.9) | 3,425 | (22.6) | 3,294 | (20.0) | 3,105 | (20.6) | 3,415 | (20.9) |
|  | 50–59 | 1,260 | (19.5) | 949 | (17.3) | 918 | (19.1) | 2,907 | (19.2) | 3,534 | (21.5) | 3,342 | (22.2) | 3,523 | (21.5) |
|  | 60–69 | 1,007 | (15.6) | 804 | (14.6) | 811 | (16.8) | 2,790 | (18.4) | 3,146 | (19.1) | 2,907 | (19.3) | 3,229 | (19.7) |
|  | ≥70 | 604 | (9.3) | 555 | (10.1) | 517 | (10.7) | 2,506 | (16.6) | 3,054 | (18.6) | 2,930 | (19.5) | 3,280 | (20.0) |
| Household income^1^ | | | |  |  |  |  |  |  |  |  |  |  |  |  |
|  | Low | 1,246 | (19.3) | 956 | (18.5) | 1,003 | (21.0) | 2,942 | (20.0) | 3,233 | (20.0) | 2,994 | (20.0) | 3,240 | (19.9) |
|  | low-Middle | 1,220 | (18.9) | 1,091 | (21.1) | 934 | (19.6) | 2,972 | (20.2) | 3,263 | (20.1) | 2,965 | (19.9) | 3,285 | (20.1) |
|  | Middle | 1,358 | (21.0) | 1,001 | (19.4) | 929 | (19.4) | 2,904 | (19.8) | 3,239 | (20.0) | 2,996 | (20.1) | 3,262 | (20.0) |
|  | Middle-high | 1,355 | (20.9) | 995 | (19.3) | 969 | (20.3) | 2,960 | (20.1) | 3,205 | (19.8) | 3,023 | (20.2) | 3,263 | (20.0) |
|  | High | 1,290 | (19.9) | 1,121 | (21.7) | 942 | (19.7) | 2,918 | (19.9) | 3,254 | (20.1) | 2,957 | (19.8) | 3,255 | (20.0) |
| Area of residence | |  |  |  |  |  |  |  |  |  |  |  |  |  |  |
|  | Urban area (Dong) | 3,970 | (61.4) | 4,151 | (75.5) | 3,629 | (75.3) | 10,806 | (71.4) | 12,773 | (77.7) | 11,883 | (79.0) | 13,132 | (80.2) |
|  | Rural area | 2,499 | (38.6) | 1,349 | (24.5) | 1,189 | (24.7) | 4,320 | (28.6) | 3,658 | (22.3) | 3,159 | (21.0) | 3,235 | (19.8) |

Values are presented as number (weighted %).

^1^Calculated as monthly household income divided by square root of the number of persons in the household, categorized into quantiles according to age and gender.

**Table 2. Trends in the prevalence of obesity among Koreans aged ≥30 years^1^**

| Variables | | | 1998 | 2001 | 2005 | 2007 | 2008 | 2009 | 2010 | 2011 | 2012 | 2013 | 2014 | 2015 | 2016 | 2017 | 2018 | Difference  (1998–2018) | APC^2^  1998–2018 | APC and year of any significant change in trend slope | | |
| --- | --- | --- | --- | --- | --- | --- | --- | --- | --- | --- | --- | --- | --- | --- | --- | --- | --- | --- | --- | --- | --- | --- |
|  |  |  |  |  |  |  |  |  |  |  |  |  |  |  |  |  |  |  |  | Before | Change year | After |
| **Total (≥30 yr)** | | | 29.1 | 32.7 | 34.8 | 34.6 | 32.9 | 34.0 | 33.9 | 34.2 | 35.4 | 34.6 | 32.9 | 36.0 | 37.0 | 35.5 | 36.9 | 7.8 | 0.9 | 1.7 | 2005 | 0.5^***^ |
| **Men** | | Total | 26.8 | 33.6 | 37.6 | 37.8 | 36.6 | 37.8 | 38.7 | 37.7 | 38.0 | 40.1 | 39.5 | 41.8 | 43.3 | 42.4 | 44.7 | 17.9 | 1.9^***^ | 3.6^***^ | 2005 | 1.4^***^ |
| Age (yr) | 30–39 | | 28.4 | 35.0 | 38.1 | 41.7 | 38.2 | 38.5 | 42.3 | 40.7 | 40.6 | 47.1 | 43.9 | 43.6 | 45.4 | 46.7 | 51.3 | 22.9 | 2.4^***^ | 3.4 | 2005 | 2.0^***^ |
|  | 40–49 | | 33.3 | 39.0 | 41.1 | 37.9 | 41.1 | 41.9 | 41.3 | 42.6 | 45.0 | 41.5 | 39.6 | 45.6 | 49.0 | 44.7 | 47.5 | 14.2 | 1.4^***^ | 1.2^***^ | 2014 | 2.4 |
|  | 50–59 | | 28.3 | 32.4 | 41.0 | 41.7 | 39.6 | 43.4 | 36.8 | 34.7 | 33.2 | 40.8 | 41.5 | 40.3 | 39.7 | 44.3 | 40.9 | 12.6 | 1.4^***^ | 4.9 | 2005 | 0.3 |
|  | 60–69 | | 20.0 | 28.0 | 31.0 | 34.6 | 29.8 | 32.2 | 37.8 | 34.1 | 33.5 | 29.3 | 36.9 | 38.3 | 39.7 | 36.7 | 38.1 | 18.1 | 2.4^***^ | 5.5 | 2005 | 1.6^***^ |
|  | ≥70 | | 8.0 | 23.0 | 27.4 | 21.4 | 21.0 | 19.9 | 24.5 | 23.7 | 23.0 | 26.2 | 24.0 | 32.1 | 30.3 | 25.3 | 30.6 | 22.6 | 2.9^***^ | 6.2 | 2005 | 2.4^***^ |
| Household income^3^ | Low | | 24.1 | 32.7 | 33.9 | 34.6 | 34.4 | 34.7 | 32.0 | 28.9 | 32.1 | 35.3 | 39.8 | 44.0 | 44.8 | 45.4 | 39.0 | 14.9 | 2.5^***^ | 1.5 | 2011 | 4.4^***^ |
|  | Low-middle | | 25.7 | 31.1 | 34.3 | 34.4 | 36.4 | 37.7 | 43.0 | 38.1 | 44.8 | 42.9 | 41.5 | 47.2 | 39.7 | 43.2 | 49.7 | 24 | 2.7^***^ | 3.6^***^ | 2010 | 1.8 |
|  | Middle | | 25.9 | 34.9 | 35.9 | 39.0 | 40.6 | 37.0 | 35.0 | 40.9 | 36.7 | 40.3 | 39.6 | 40.4 | 44.8 | 38.1 | 43.5 | 17.6 | 1.8^***^ | 4.2 | 2005 | 0.9 |
|  | Middle-high | | 27.3 | 34.1 | 40.5 | 45.0 | 35.0 | 38.3 | 39.2 | 44.7 | 37.7 | 37.2 | 39.3 | 39.2 | 40.9 | 46.1 | 49.5 | 22.2 | 1.9^***^ | 1.5^***^ | 2016 | 8.7 |
|  | High | | 30.5 | 35.0 | 42.1 | 38.7 | 39.0 | 41.2 | 44.1 | 36.9 | 40.1 | 45.4 | 37.5 | 40.4 | 45.8 | 39.8 | 41.3 | 10.8 | 1.2^***^ | 4.0 | 2005 | 0.2 |
| **Women** | | Total | 30.5 | 32.1 | 31.3 | 30.3 | 28.4 | 29.5 | 28.5 | 30.1 | 32.2 | 28.2 | 25.7 | 29.6 | 30.0 | 27.7 | 28.3 | -2.2 | -0.5^***^ | -0.3 | 2005 | -0.6 |
| Age (yr) | 30–39 | | 20.9 | 19.1 | 19.0 | 12.8 | 17.0 | 19.6 | 19.0 | 21.7 | 23.7 | 17.9 | 18.6 | 21.1 | 21.7 | 18.3 | 22.6 | 1.7 | 0.2 | -1.8 | 2007 | 2.0^***^ |
|  | 40–49 | | 29.8 | 33.6 | 29.0 | 26.6 | 27.5 | 27.2 | 26.7 | 27.9 | 33.2 | 25.7 | 22.3 | 25.4 | 28.7 | 25.6 | 25.7 | -4.1 | -1.0^***^ | -0.8 | 2012 | -1.3 |
|  | 50–59 | | 42.7 | 40.8 | 43.1 | 43.1 | 35.3 | 36.7 | 33.8 | 36.7 | 34.9 | 33.7 | 29.3 | 36.2 | 32.5 | 31.7 | 29.3 | -13.4 | -1.8^***^ | -0.4 | 2005 | -2.3^***^ |
|  | 60–69 | | 38.6 | 46.6 | 47.1 | 56.4 | 43.8 | 41.4 | 43.3 | 43.0 | 43.1 | 42.7 | 36.6 | 41.7 | 40.7 | 39.3 | 35.5 | -3.1 | -0.8 | 2.7 | 2005 | -2.0^***^ |
|  | ≥70 | | 29.4 | 33.4 | 34.0 | 37.9 | 34.3 | 38.1 | 34.4 | 33.5 | 36.1 | 38.6 | 37.3 | 40.8 | 42.2 | 41.0 | 43.0 | 13.6 | 1.7^***^ | 1.0^***^ | 2011 | 2.7^***^ |
| Household income^3^ | Low | | 30.1 | 36.0 | 34.3 | 29.5 | 35.9 | 35.0 | 32.3 | 37.1 | 38.4 | 33.2 | 29.9 | 41.6 | 37.8 | 36.1 | 35.5 | 5.4 | 0.7 | 0.9 | 2016 | -2.4 |
|  | Low-Middle | | 31.1 | 35.7 | 34.4 | 32.4 | 31.9 | 30.8 | 28.1 | 29.2 | 36.8 | 32.5 | 27.1 | 29.1 | 31.8 | 30.6 | 32.3 | 1.2 | -0.4 | -0.6 | 2015 | 1.7 |
|  | Middle | | 33.0 | 32.5 | 31.8 | 32.0 | 28.4 | 31.5 | 30.0 | 33.8 | 27.9 | 28.6 | 29.6 | 28.0 | 29.3 | 26.7 | 30.9 | -2.1 | -0.7^***^ | -0.8^***^ | 2016 | 2.0 |
|  | Middle-high | | 30.0 | 30.1 | 31.4 | 26.4 | 25.8 | 25.7 | 28.7 | 22.6 | 29.7 | 26.0 | 25.6 | 29.6 | 27.6 | 24.2 | 22.3 | -7.7 | -1.0^***^ | -0.7 | 2016 | -8.0 |
|  | High | | 28.0 | 27.3 | 24.9 | 30.1 | 20.9 | 24.9 | 23.6 | 26.0 | 26.0 | 19.7 | 17.1 | 20.5 | 23.8 | 21.1 | 18.0 | -10 | -1.8^***^ | -1.0 | 2007 | -2.3^***^ |

Values are presented as weighted %.

^1^Age-standardized prevalence was calculated using the 2005 Population Projections for Korea.

^2^The annual percent change (APC) is significantly different from 0.

^3^Calculated as monthly household income divided by square root of the number of persons in the household, categorized into quantiles according to age and gender.

^***^p*<*0.001

**Table 3. Trends in the prevalence of hypertension among Koreans aged ≥30 years^1^**

| Variables | | | 1998 | 2001 | 2005 | 2007 | 2008 | 2009 | 2010 | 2011 | 2012 | 2013 | 2014 | 2015 | 2016 | 2017 | 2018 | Difference (1998–2018) | APC^2^  1998–2018 | APC and year of any significant change in trend slope | | |
| --- | --- | --- | --- | --- | --- | --- | --- | --- | --- | --- | --- | --- | --- | --- | --- | --- | --- | --- | --- | --- | --- | --- |
|  |  |  |  |  |  |  |  |  |  |  |  |  |  |  |  |  |  |  |  | Before | Change year | After |
| **Total (≥30 yr)** | | | 29.8 | 28.5 | 28.0 | 24.5 | 26.2 | 26.3 | 26.8 | 28.4 | 28.9 | 27.2 | 25.4 | 27.8 | 29.1 | 26.9 | 28.3 | -1.5 | -0.2 | -1.3 | 2007 | 0.6 |
| **Men** | | Total | 32.4 | 33.2 | 31.5 | 26.8 | 28.1 | 30.3 | 29.3 | 32.8 | 32.1 | 32.4 | 29.7 | 32.6 | 35.0 | 32.3 | 33.2 | 0.8 | 0.3 | -1.1 | 2008 | 1.4^***^ |
| Age (yr) | 30–39 | | 18.6 | 17.6 | 14.1 | 13.3 | 15.2 | 11.5 | 12.6 | 14.6 | 15.5 | 15.8 | 13.6 | 15.9 | 16.9 | 17.9 | 17.1 | -1.5 | -0.6 | -3.3^***^ | 2009 | 3.5^***^ |
|  | 40–49 | | 30.5 | 28.6 | 27.8 | 19.8 | 23.5 | 25.2 | 22.4 | 31.2 | 26.9 | 28.5 | 26.9 | 28.4 | 30.8 | 26.9 | 29.1 | -1.4 | 0.0 | -2.6 | 2007 | 1.8^***^ |
|  | 50–59 | | 42.0 | 40.0 | 44.1 | 36.0 | 35.9 | 40.2 | 41.0 | 38.0 | 38.7 | 41.3 | 36.8 | 38.4 | 42.3 | 39.4 | 40.2 | -1.8 | -0.2 | -0.9 | 2008 | 0.3 |
|  | 60–69 | | 43.8 | 56.8 | 53.9 | 43.4 | 43.0 | 52.1 | 52.5 | 53.5 | 55.3 | 48.5 | 45.5 | 52.1 | 55.9 | 47.6 | 47.5 | 3.7 | 0.1 | 0.5 | 2016 | -5.8 |
|  | ≥70 | | 48.8 | 52.5 | 43.9 | 51.9 | 48.4 | 58.5 | 50.1 | 58.9 | 58.1 | 59.0 | 55.8 | 61.7 | 64.2 | 61.6 | 65.1 | 16.3 | 1.7^***^ | -0.2 | 2005 | 2.1^***^ |
| Household income^3^ | Low | | 34.8 | 37.2 | 35.8 | 32.8 | 29.9 | 29.0 | 28.7 | 31.2 | 30.5 | 33.3 | 28.1 | 39.2 | 38.3 | 35.0 | 35.4 | 0.6 | 0.1 | -1.8^***^ | 2010 | 3.0^***^ |
|  | Low-middle | | 33.2 | 31.1 | 31.2 | 28.7 | 28.7 | 33.0 | 29.3 | 31.7 | 33.5 | 31.1 | 31.6 | 32.3 | 31.1 | 32.4 | 37.6 | 4.4 | 0.4 | -0.1 | 2016 | 8.9 |
|  | Middle | | 31.1 | 33.8 | 26.8 | 26.6 | 28.1 | 27.3 | 31.2 | 39.5 | 33.2 | 33.5 | 28.7 | 29.2 | 32.6 | 27.4 | 30.4 | -0.7 | 0.0 | 0.6 | 2012 | -1.7 |
|  | Middle-high | | 30.3 | 33.0 | 30.8 | 25.5 | 27.1 | 30.7 | 29.1 | 29.3 | 34.5 | 31.7 | 32.7 | 31.9 | 38.1 | 32.7 | 32.1 | 1.8 | 0.7 | -1.1 | 2007 | 1.9^***^ |
|  | High | | 33.3 | 30.4 | 32.5 | 23.9 | 26.5 | 31.0 | 27.9 | 31.2 | 29.5 | 31.7 | 27.5 | 30.8 | 35.2 | 33.9 | 30.0 | -3.3 | 0.1 | -1.8 | 2007 | 1.4^***^ |
| **Women** | | Total | 26.8 | 25.3 | 23.8 | 21.7 | 23.8 | 22.1 | 23.8 | 23.6 | 25.2 | 22.1 | 20.9 | 22.9 | 22.9 | 21.3 | 23.1 | -3.7 | -0.9^***^ | -1.7 | 2005 | -0.5 |
| Age (yr) | 30–39 | | 6.2 | 5.4 | 3.1 | 1.5 | 4.3 | 2.9 | 1.6 | 3.4 | 3.2 | 3.7 | 1.1 | 1.6 | 3.3 | 4.2 | 5.8 | -0.4 | -2.7 | -6.0^***^ | 2015 | 40.4 |
|  | 40–49 | | 19.6 | 15.2 | 11.2 | 11.4 | 15.0 | 11.0 | 11.6 | 10.8 | 18.1 | 10.7 | 8.8 | 13.0 | 12.4 | 11.3 | 11.9 | -7.7 | -2.4^***^ | -5.8 | 2005 | -0.5 |
|  | 50–59 | | 37.2 | 33.2 | 38.4 | 31.1 | 32.6 | 30.5 | 33.7 | 29.7 | 30.4 | 30.6 | 27.4 | 29.5 | 30.8 | 24.9 | 29.1 | -8.1 | -1.4^***^ | -0.7 | 2005 | -1.8^***^ |
|  | 60–69 | | 50.5 | 57.5 | 53.6 | 48.2 | 50.2 | 49.1 | 58.3 | 57.1 | 52.8 | 48.8 | 51.1 | 51.5 | 46.2 | 46.3 | 44.6 | -5.9 | -0.6 | 0.3 | 2011 | -2.5^***^ |
|  | ≥70 | | 63.4 | 61.5 | 61.3 | 65.4 | 61.5 | 65.9 | 68.2 | 71.5 | 71.6 | 64.3 | 68.7 | 71.3 | 72.5 | 66.8 | 73.7 | 10.3 | 0.9^***^ | 0.1 | 2005 | 1.1^***^ |
| Household income^3^ | Low | | 27.8 | 25.4 | 25.8 | 24.9 | 26.0 | 23.8 | 26.6 | 24.0 | 26.2 | 21.8 | 24.6 | 24.9 | 27.5 | 24.8 | 25.9 | -1.9 | -0.3 | -0.8^***^ | 2013 | 1.6 |
|  | Low-middle | | 25.7 | 25.1 | 24.4 | 20.7 | 24.3 | 21.6 | 22.7 | 26.2 | 27.6 | 23.9 | 24.8 | 28.8 | 21.5 | 22.6 | 26.5 | 0.8 | -0.1 | -1.1 | 2007 | 0.7 |
|  | Middle | | 29.7 | 26.2 | 23.8 | 22.1 | 25.0 | 22.9 | 23.0 | 23.9 | 26.0 | 26.7 | 19.2 | 20.0 | 21.5 | 23.4 | 23.5 | -6.2 | -1.2^***^ | -1.5^***^ | 2016 | 5.0 |
|  | Middle-high | | 25.9 | 26.5 | 23.4 | 19.2 | 21.5 | 19.7 | 23.0 | 22.2 | 22.8 | 18.5 | 18.1 | 20.6 | 22.7 | 17.5 | 22.3 | -3.6 | -1.4^***^ | -1.8^***^ | 2014 | 1.8 |
|  | High | | 25.1 | 23.3 | 22.1 | 22.3 | 22.3 | 21.8 | 24.5 | 21.1 | 22.4 | 20.0 | 18.4 | 20.4 | 21.0 | 18.8 | 17.4 | -7.7 | -1.4^***^ | -1.1^***^ | 2016 | -7.1 |

Values are presented as weighted %.

^1^Age-standardized prevalence was calculated using the 2005 Population Projections for Korea.

^2^The annual percent change (APC) is significantly different from 0.

^3^Calculated as monthly household income divided by square root of the number of persons in the household, categorized into quantiles according to age and gender.

^***^p*<*0.001

**Table 4. Trends in the awareness, treatment, and control rates of hypertension, diabetes, and hypercholesterolemia among Koreans ≥30 years**

| Variables | | | 1998 | 2001 | 2005 | 2007-2009 | 2010-2012 | 2013-2015 | 2016-2018 | Difference  (1998 to 2018) | APC ^1^  1998-2018 |
| --- | --- | --- | --- | --- | --- | --- | --- | --- | --- | --- | --- |
| Hypertension | Awareness | Total | 23.5 | 34.1 | 57.1 | 66.3 | 65.9 | 67.3 | 69.1 | 45.6 | 9.2^***^ |
|  |  | Men | 17.3 | 26.8 | 48.4 | 58.4 | 56.9 | 60.1 | 64.0 | 46.7 | 12.1^***^ |
|  |  | Women | 30.0 | 40.8 | 67.2 | 75.0 | 75.7 | 75.4 | 75.2 | 45.2 | 6.1 |
|  | Treatment | Total | 20.4 | 32.7 | 49.6 | 60.3 | 60.7 | 63.6 | 65.3 | 44.9 | 11.1^***^ |
|  |  | Men | 13.8 | 25.2 | 39.4 | 51.1 | 51.4 | 55.4 | 59.7 | 45.9 | 14.3^***^ |
|  |  | Women | 27.5 | 39.5 | 61.4 | 70.3 | 70.7 | 72.7 | 72.0 | 44.5 | 7.5^***^ |
|  | Control | Total | 23.8 | 37.6 | 54.9 | 69.3 | 69.1 | 72.0 | 73.1 | 49.3 | 6.2^***^ |
|  |  | Men | 23.5 | 30.2 | 51.1 | 68.7 | 71.8 | 72.4 | 75.8 | 52.3 | 7.0^***^ |
|  |  | Women | 24.0 | 42.0 | 57.8 | 69.7 | 67.0 | 71.7 | 70.5 | 46.5 | 4.6 |
| Diabetes | Awareness | Total | - | - | 68.3 | 72.6 | 72.7 | 70.5 | 71.5 | 3.2 | 0.0 |
|  |  | Men | - | - | 67.0 | 70.2 | 69.5 | 66.6 | 66.7 | -0.3 | -1.5 |
|  |  | Women | - | - | 69.9 | 75.5 | 76.7 | 75.5 | 77.7 | 7.8 | 1.6^***^ |
|  | Treatment | Total | - | - | 49.0 | 57.5 | 63.9 | 63.3 | 66.2 | 17.2 | 4.9^***^ |
|  |  | Men | - | - | 44.8 | 53.6 | 60.8 | 59.1 | 61.7 | 16.9 | 4.7^***^ |
|  |  | Women | - | - | 54.2 | 62.0 | 67.7 | 68.9 | 71.9 | 17.7 | 5.5^***^ |
|  | Control | Total | - | - | 22.0 | 24.6 | 25.0 | 22.3 | 25.8 | 3.8 | 0.7 |
|  |  | Men | - | - | 25.1 | 27.1 | 23.7 | 23.2 | 25.1 | 0.0 | -2.0 |
|  |  | Women | - | - | 19.0 | 22.0 | 26.5 | 21.3 | 26.5 | 7.5 | 3.5 |
| Hypercholesterolemia | Awareness | Total | - | - | 24.0 | 38.8 | 47.4 | 57.7 | 60.1 | 36.1 | 16.1^***^ |
|  |  | Men | - | - | 24.4 | 37.0 | 45.2 | 51.4 | 57.3 | 32.9 | 15.3^***^ |
|  |  | Women | - | - | 23.8 | 40.3 | 49.1 | 62.4 | 62.4 | 38.6 | 16.2^***^ |
|  | Treatment | Total | - | - | 17.3 | 26.9 | 37.3 | 45.5 | 50.3 | 33.0 | 22.0^***^ |
|  |  | Men | - | - | 17.5 | 24.6 | 35.8 | 39.8 | 48.8 | 31.3 | 22.4^***^ |
|  |  | Women | - | - | 17.1 | 28.6 | 38.4 | 49.9 | 51.5 | 34.4 | 21.3^***^ |
|  | Control | Total | - | - | 62.3 | 73.5 | 78.8 | 84.3 | 84.0 | 21.7 | 4.0^***^ |
|  |  | Men | - | - | 63.1 | 80.6 | 79.3 | 88.4 | 85.7 | 22.6 | 2.7 |
|  |  | Women | - | - | 61.8 | 68.8 | 78.5 | 81.8 | 82.7 | 20.9 | 4.5^***^ |

Values are presented as weighted %; all estimates are crude.

^1^The annual percent change (APC) is significantly different from 0.

^***^p*<*0.001

**Table 5. Trends in the prevalence of diabetes among Koreans aged ≥30 years^1^**

| Variables | | | 2005 | 2007 | 2008 | 2009 | 2010 | 2011 | 2012 | 2013 | 2014 | 2015 | 2016 | 2017 | 2018 | Difference  (2005–2018) | APC^2^  2005–2018 | APC and year of any significant change in trend slope | | |
| --- | --- | --- | --- | --- | --- | --- | --- | --- | --- | --- | --- | --- | --- | --- | --- | --- | --- | --- | --- | --- |
|  |  |  |  |  |  |  |  |  |  |  |  |  |  |  |  |  |  | Before | Change year | After |
| **Total (≥30 yr)** | | | 9.1 | 9.5 | 9.7 | 9.6 | 9.6 | 9.7 | 9.0 | 11.0 | 10.1 | 9.5 | 11.3 | 10.4 | 10.4 | 1.3 | 1.2^***^ | 1.4^***^ | 2016 | -1.0 |
| **Men** | | Total | 10.5 | 11.8 | 10.6 | 10.7 | 11.0 | 11.9 | 10.1 | 12.8 | 12.5 | 11.0 | 12.9 | 12.4 | 12.9 | 2.4 | 1.6^***^ | 0.4 | 2009 | 1.9^***^ |
| Age (yr) | 30–39 | | 1.7 | 6.7 | 2.5 | 2.8 | 3.3 | 3.7 | 1.7 | 3.7 | 2.0 | 2.8 | 3.6 | 3.0 | 2.7 | 1.0 | -1.0 | 7.1 | 2008 | -1.8 |
|  | 40–49 | | 9.3 | 6.9 | 10.2 | 6.9 | 8.4 | 8.5 | 4.4 | 10.2 | 10.0 | 9.5 | 9.8 | 8.4 | 11.7 | 2.4 | 1.4 | -2.6 | 2012 | 6.0 |
|  | 50–59 | | 18.9 | 16.8 | 15.2 | 15.7 | 17.7 | 19.5 | 15.6 | 16.3 | 15.8 | 12.6 | 17.4 | 19.1 | 16.2 | -2.7 | -0.2 | -1.5 | 2015 | 6.0 |
|  | 60–69 | | 17.9 | 26.1 | 21.2 | 25.2 | 16.0 | 23.4 | 25.6 | 28.5 | 30.0 | 22.4 | 25.5 | 23.1 | 24.6 | 6.7 | 1.6 | 4.0 | 2014 | -3.7 |
|  | ≥70 | | 16.7 | 14.4 | 15.1 | 18.9 | 24.5 | 18.2 | 22.8 | 22.5 | 24.4 | 23.5 | 26.4 | 27.7 | 27.7 | 11.0 | 4.8^***^ | 8.1^***^ | 2010 | 3.9^***^ |
| Household income^3^ | Low | | 9.8 | 10.3 | 15.3 | 12.5 | 13.9 | 12.5 | 9.7 | 15.3 | 17.0 | 14.0 | 16.0 | 17.7 | 15.3 | 5.5 | 3.2^***^ | 9.8 | 2008 | 2.5 |
|  | Low-middle | | 10.0 | 9.1 | 9.4 | 10.3 | 11.9 | 10.6 | 11.3 | 14.7 | 12.4 | 15.5 | 12.1 | 11.2 | 16.3 | 6.3 | 3.3^***^ | 4.2^***^ | 2015 | -0.3 |
|  | Middle | | 11.1 | 13.2 | 10.5 | 12.0 | 8.4 | 12.7 | 7.6 | 10.1 | 9.6 | 8.4 | 12.9 | 13.2 | 10.6 | -0.5 | 0.6 | -2.1 | 2014 | 7.9 |
|  | Middle-high | | 9.9 | 15.5 | 7.6 | 10.2 | 8.9 | 9.3 | 9.5 | 13.2 | 10.8 | 7.0 | 11.8 | 11.2 | 11.6 | 1.7 | 0.7 | -3.0 | 2010 | 2.4 |
|  | High | | 12.2 | 11.1 | 10.9 | 8.0 | 11.0 | 14.0 | 12.3 | 11.2 | 13.4 | 10.7 | 11.0 | 10.1 | 10.9 | -1.3 | -0.2 | 1.0 | 2014 | -3.3 |
| **Women** | | Total | 7.6 | 7.2 | 8.5 | 8.4 | 8.2 | 7.5 | 8.0 | 9.0 | 7.9 | 8.0 | 9.6 | 8.4 | 7.9 | 0.3 | 0.5 | 1.1 | 2016 | -4.5 |
| Age (yr) | 30–39 | | 1.2 | 1.5 | 1.7 | 2.3 | 2.3 | 1.3 | 2.0 | 1.2 | 2.4 | 3.0 | 1.8 | 0.9 | 2.2 | 1.0 | 1.6 | 16.0 | 2009 | -1.2 |
|  | 40–49 | | 5.3 | 4.4 | 4.9 | 4.7 | 4.8 | 4.6 | 5.8 | 4.4 | 4.9 | 4.5 | 6.1 | 5.0 | 3.3 | -2.0 | -0.9 | 0.9 | 2016 | -17.5 |
|  | 50–59 | | 9.1 | 9.9 | 9.9 | 8.9 | 8.6 | 8.2 | 9.5 | 9.0 | 8.1 | 6.9 | 11.0 | 11.0 | 9.8 | 0.7 | 0.8 | -1.3 | 2014 | 6.2 |
|  | 60–69 | | 18.3 | 14.5 | 21.1 | 19.2 | 19.1 | 16.0 | 15.5 | 22.1 | 16.1 | 17.1 | 18.3 | 16.2 | 16.3 | -2.0 | -1.2 | 3.2 | 2008 | -1.7 |
|  | ≥70 | | 18.5 | 19.3 | 21.9 | 23.8 | 22.7 | 23.7 | 21.5 | 31.3 | 23.6 | 25.0 | 30.9 | 28.1 | 25.7 | 7.2 | 2.8^***^ | 3.8^***^ | 2016 | -4.4 |
| Household income^3^ | Low | | 6.1 | 10.5 | 11.0 | 8.7 | 8.7 | 8.0 | 10.9 | 11.8 | 11.6 | 11.4 | 11.8 | 11.5 | 10.6 | 4.5 | 2.8^***^ | 14.6 | 2008 | 1.7 |
|  | Low-middle | | 8.2 | 9.8 | 9.4 | 8.4 | 10.4 | 7.5 | 11.8 | 8.7 | 8.0 | 8.9 | 9.7 | 9.3 | 9.6 | 1.4 | 0.4 | 3.2 | 2008 | 0.1 |
|  | Middle | | 7.7 | 7.0 | 7.5 | 8.5 | 8.6 | 7.8 | 5.6 | 7.9 | 5.2 | 6.8 | 10.8 | 7.7 | 7.7 | 0.0 | 0.5 | -1.6 | 2014 | 6.2 |
|  | Middle-high | | 8.8 | 3.3 | 7.1 | 7.3 | 6.5 | 7.9 | 6.9 | 8.6 | 7.1 | 5.7 | 6.6 | 7.1 | 6.4 | -2.4 | -1.2 | -5.1 | 2008 | -0.6 |
|  | High | | 7.4 | 5.3 | 6.2 | 8.9 | 7.2 | 6.5 | 3.4 | 8.2 | 7.6 | 7.6 | 8.5 | 6.7 | 5.2 | -2.2 | -0.1 | 1.7 | 2016 | -17.5 |

Values are presented as weighted %.

^1^Age-standardized prevalence was calculated using the 2005 Population Projections for Korea.

^2^The annual percent change (APC) is significantly different from 0.

^3^Calculated as monthly household income divided by square root of the number of persons in the household, categorized into quantiles according to age and gender.

^***^p*<*0.001

**Table 6. Trends in the prevalence of hypercholesterolemia among Koreans aged ≥30 years^1^**

| Variables | | | 2005 | 2007 | 2008 | 2009 | 2010 | 2011 | 2012 | 2013 | 2014 | 2015 | 2016 | 2017 | 2018 | Difference  (2005–2018) | APC ^2^  2005–2018 | APC and year of any significant change in trend slope | | | | |
| --- | --- | --- | --- | --- | --- | --- | --- | --- | --- | --- | --- | --- | --- | --- | --- | --- | --- | --- | --- | --- | --- | --- |
|  |  |  |  |  |  |  |  |  |  |  |  |  |  |  |  |  |  | Before | Change year | | After | |
| **Total (≥30 yr)** | | | 8.0 | 10.7 | 10.8 | 11.4 | 13.4 | 13.8 | 14.4 | 14.9 | 14.6 | 17.9 | 19.9 | 21.5 | 21.4 | 13.4 | 7.6^***^ | 10.4 | | 2008 | | 7.3^***^ |
| **Men** | | Total | 7.3 | 9.3 | 9.5 | 10.8 | 13.0 | 12.6 | 12.2 | 13.6 | 13.9 | 16.4 | 19.3 | 20.0 | 20.9 | 13.6 | 8.2^***^ | 7.4^***^ | | 2014 | | 9.9^***^ |
| Age (yr) | 30–39 | | 5.9 | 7.5 | 7.5 | 8.4 | 8.9 | 10.3 | 11.3 | 11.9 | 7.6 | 10.6 | 10.8 | 14.8 | 15.1 | 9.2 | 6.4^***^ | 5.4^***^ | | 2016 | | 15.0 |
|  | 40–49 | | 8.7 | 11.0 | 10.5 | 11.0 | 14.3 | 10.4 | 8.1 | 12.1 | 16.3 | 18.6 | 20.2 | 18.3 | 22.9 | 14.2 | 7.7^***^ | 2.7 | | 2012 | | 12.4^***^ |
|  | 50–59 | | 7.9 | 10.9 | 10.9 | 14.0 | 18.7 | 16.2 | 16.0 | 13.5 | 18.3 | 16.2 | 28.1 | 24.8 | 20.8 | 12.9 | 7.3^***^ | 15.0 | | 2010 | | 5.5^***^ |
|  | 60–69 | | 8.8 | 9.6 | 10.5 | 12.8 | 13.3 | 17.0 | 16.6 | 18.8 | 20.3 | 23.2 | 27.2 | 25.8 | 27.0 | 18.2 | 9.4^***^ | 10.9^***^ | | 2016 | | 1.3 |
|  | ≥70 | | 3.6 | 6.6 | 8.4 | 8.5 | 10.6 | 13.5 | 14.5 | 16.8 | 9.7 | 20.1 | 16.4 | 25.3 | 25.7 | 22.1 | 12.5^***^ | 32.8 | | 2008 | | 11.7^***^ |
| Household income^3^ | Low | | 8.6 | 8.4 | 12.5 | 10.8 | 14.1 | 13.2 | 10.8 | 13.2 | 10.9 | 15.3 | 21.0 | 23.7 | 18.9 | 10.3 | 7.1^***^ | 4.2 | | 2014 | | 12.7 |
|  | Low-Middle | | 6.3 | 6.5 | 6.9 | 8.6 | 11.8 | 12.3 | 14.7 | 10.6 | 12.9 | 16.2 | 16.3 | 16.9 | 21.2 | 14.9 | 9.1^***^ | 13.1^***^ | | 2011 | | 7.4^***^ |
|  | Middle | | 7.7 | 11.5 | 9.4 | 12.7 | 13.2 | 13.6 | 9.7 | 14.1 | 15.1 | 15.4 | 18.8 | 19.6 | 23.1 | 15.4 | 7.4^***^ | 5.7^***^ | | 2015 | | 13.2 |
|  | Middle-high | | 6.5 | 8.0 | 7.8 | 9.8 | 10.8 | 10.0 | 11.5 | 17.1 | 13.6 | 16.2 | 20.3 | 17.2 | 20.9 | 14.4 | 9.5^***^ | 10.3^***^ | | 2016 | | 3.0 |
|  | High | | 6.8 | 11.7 | 10.2 | 11.9 | 13.2 | 13.9 | 15.8 | 13.5 | 17.4 | 17.5 | 19.5 | 23.3 | 20.4 | 13.6 | 8.0^***^ | 13.5^***^ | | 2009 | | 7.1^***^ |
| **Women** | | Total | 8.4 | 11.5 | 11.8 | 11.8 | 13.4 | 14.8 | 16.3 | 15.9 | 14.9 | 19.1 | 20.2 | 22.6 | 21.4 | 13.0 | 7.1^***^ | 11.3 | | 2008 | | 6.8^***^ |
| Age (yr) | 30–39 | | 1.8 | 3.5 | 4.3 | 4.1 | 3.1 | 5.0 | 5.7 | 4.9 | 4.4 | 5.8 | 8.0 | 7.7 | 9.2 | 7.4 | 9.8^***^ | 26.0 | | 2008 | | 8.5^***^ |
|  | 40–49 | | 5.5 | 5.7 | 6.5 | 7.7 | 6.3 | 9.6 | 10.4 | 8.0 | 9.1 | 12.6 | 9.6 | 17.9 | 10.5 | 5.0 | 7.4^***^ | 8.0^***^ | | 2016 | | 2.5 |
|  | 50–59 | | 15.2 | 19.1 | 19.5 | 21.0 | 23.3 | 22.4 | 28.0 | 26.8 | 19.6 | 28.5 | 33.7 | 31.7 | 31.9 | 16.7 | 5.5^***^ | 6.1^***^ | | 2016 | | 0.9 |
|  | 60–69 | | 17.4 | 26.5 | 24.9 | 22.5 | 29.5 | 35.8 | 31.2 | 34.5 | 32.8 | 40.6 | 47.3 | 41.4 | 44.3 | 26.9 | 6.6^***^ | 7.8^***^ | | 2016 | | -1.5 |
|  | ≥70 | | 13.2 | 20.5 | 20.2 | 17.6 | 27.5 | 19.8 | 26.4 | 29.0 | 34.0 | 34.4 | 29.0 | 42.6 | 42.9 | 29.7 | 8.6^***^ | 8.0^***^ | | 2016 | | 11.9 |
| Household income^3^ | Low | | 8.4 | 13.5 | 10.9 | 12.4 | 15.5 | 15.3 | 15.2 | 16.3 | 16.4 | 20.4 | 21.1 | 22.7 | 21.3 | 12.9 | 7.0^***^ | 11.1^***^ | | 2010 | | 5.9^***^ |
|  | Low-middle | | 8.0 | 10.2 | 10.5 | 12.9 | 11.9 | 13.6 | 17.5 | 15.5 | 15.3 | 17.5 | 17.9 | 25.2 | 24.8 | 16.8 | 8.5^***^ | 7.3^***^ | | 2016 | | 16.7 |
|  | Middle | | 8.0 | 11.8 | 10.8 | 11.4 | 11.0 | 13.5 | 14.6 | 15.9 | 15.7 | 19.9 | 20.1 | 22.1 | 21.3 | 13.3 | 7.9^***^ | 8.6^***^ | | 2016 | | 3.4 |
|  | Middle-high | | 9.4 | 8.8 | 13.0 | 11.2 | 12.4 | 16.2 | 18.7 | 14.3 | 13.7 | 18.9 | 19.6 | 20.3 | 19.6 | 10.2 | 6.0^***^ | 8.9 | | 2011 | | 4.6 |
|  | High | | 8.6 | 13.4 | 11.5 | 11.3 | 16.3 | 15.9 | 15.8 | 17.6 | 13.8 | 18.3 | 22.2 | 23.2 | 19.5 | 10.9 | 6.3^***^ | 7.5^***^ | | 2016 | | -1.5 |

Values are presented as weighted %.

^1^Age-standardized prevalence was calculated using the 2005 Population Projections for Korea.

^2^The annual percent change (APC) is significantly different from 0.

^3^Calculated as monthly household income divided by square root of the number of persons in the household, categorized into quantiles according to age and gender.

^***^p*<*0.001
